# Effectiveness of wearable technology‐based physical activity interventions for adults with type 2 diabetes mellitus: A systematic review and meta‐regression

**DOI:** 10.1111/1753-0407.70002

**Published:** 2024-10-04

**Authors:** Rachael Ern Ching Chua, Ying Lau, Wen Wei Ang, Allison Ann Ying Faustina Boey, Siew Tiang Lau

**Affiliations:** ^1^ Changi General Hospital, Department of Nursing, SingHealth System Singapore Singapore; ^2^ The Nethersole School of Nursing, Faculty of Medicine The Chinese University of Hong Kong Hong Kong, SAR Hong Kong; ^3^ Alice Lee Centre for Nursing Studies, Yong Loo Lin School of Medicine National University of Singapore Singapore Singapore

**Keywords:** meta‐analysis, meta‐regression, physical activity interventions, type 2 diabetes mellitus, wearable technology

## Abstract

Type 2 diabetes mellitus (T2DM) is a chronic metabolic disorder with the increasing prevalence of a modern sedentary lifestyle. Wearable technology‐based physical activity interventions (WT‐BPAI) might provide a channel to improve diabetic self‐management. The study aimed to (1) evaluate the effectiveness of WT‐BPAI on PA levels, glycemic levels, and other outcomes (blood pressure [BP], body mass index [BMI], and serum lipid profile) in adults with T2DM, and (2) investigate the potential covariates affecting aforementioned outcomes. Eight databases were searched thoroughly using three steps from inception until January 16, 2024. The quality of the studies and overall evidence were evaluated. The package *meta* of the *R* software program version 4.3.1. was utilized for meta‐analyses, subgroup analyses, and meta‐regression analyses. A total of 19 randomized controlled trials (RCTs) were found. Meta‐analyses revealed that WT‐BPAI significantly increased 1583 steps per day and decreased systolic BP (SBP) by 2.46 mmHg. Subgroup and meta‐regression analyses found that function, duration of intervention, and age were significant covariates. According to the risk of bias version 2, more than half of the trials raised some concerns about the randomization process, deviations from the intended intervention, and missing outcome data. The certainty of the evidence was very low for all outcomes based on the Grading of Recommendations Assessment, Development and Evaluation (GRADE) criteria. WT‐BPAI can be considered a supplementary intervention to increase the steps per day and decrease SBP, especially when used for short periods in young adults with T2DM. However, we need more well‐designed research with long‐term outcomes.

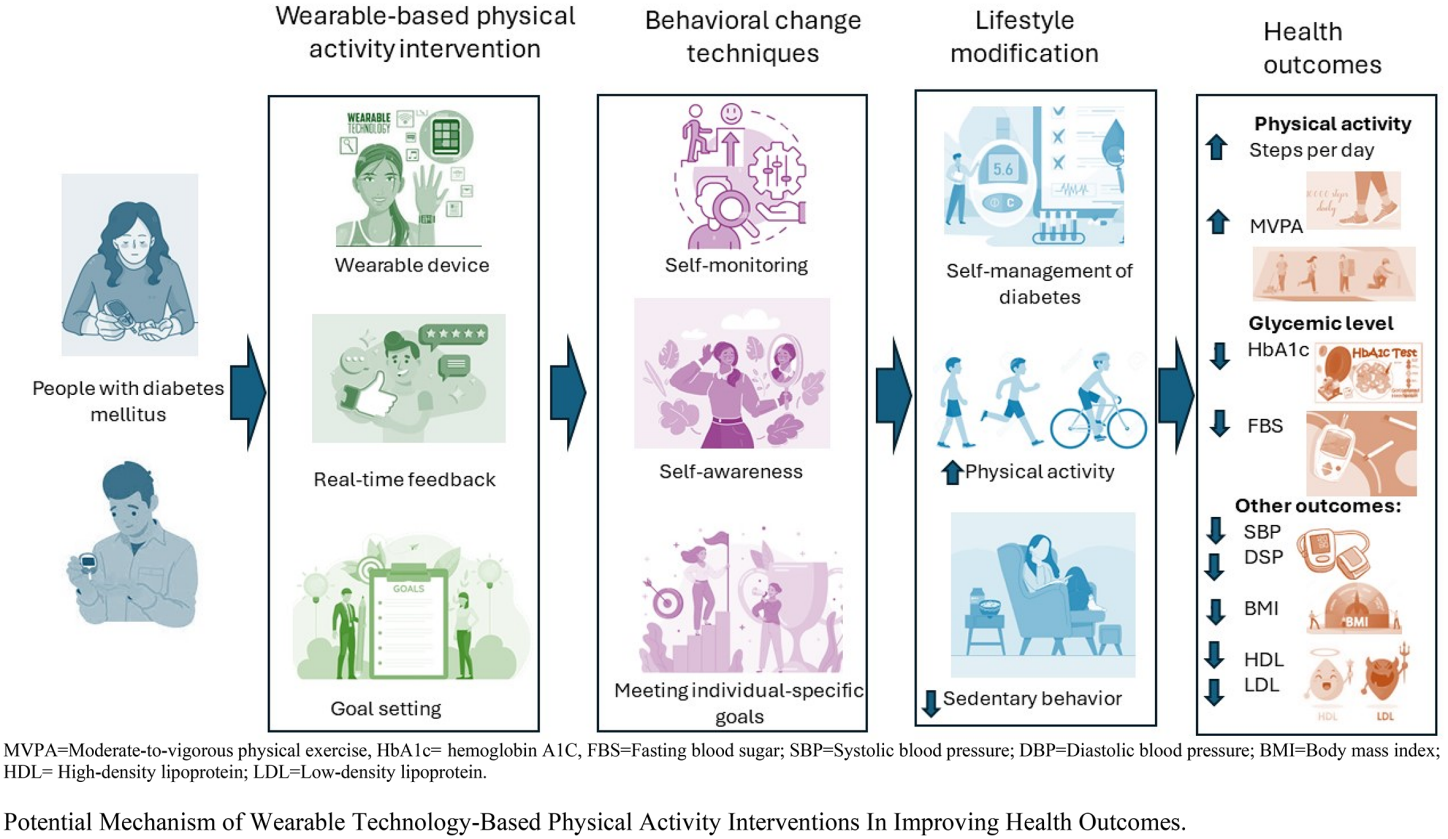

## Graphical abstract

Highlights

- In people with diabetes mellitus, wearable technology‐based physical activity interventions significantly increased daily steps and reduced systolic blood pressure.
- Intervention had a goal‐setting component among T2DM patients, which had a greater effect on lowering BMI than their counterparts.
- In terms of BMI reduction, the year of publication was a significant covariate.

## 1 INTRODUCTION

Type 2 diabetes mellitus (T2DM) is a chronic metabolic disorder that accounts for roughly 90% of all cases of diabetes mellitus (DM).^1^ T2DM occurs when there is insufficient insulin production by the pancreas, or when the insulin produced can no longer be used effectively, causing hyperglycemia.^2^ Its prevalence continues to increase rapidly with the increasing prevalence of a modern sedentary lifestyle,^3^, ^4^ leading to increased socioeconomic costs.^4^ Physical activity (PA) leads to significant glycemic level reductions,^5^ providing a channel where interventions can be implemented to improve outcomes in adults with T2DM. Although it is well established that PA is crucial in DM self‐management, and is a major modifiable risk factor, it is also notable that patients still struggle with adhering to regular exercise routines due to various reasons, including a perception that diet and medication adherence are more important,^6^ the sheer amount of effort required to change lifestyle habits,^7^ and a lack of motivation.^8^

Wearable technology‐based PA interventions (WT‐BPAI) may improve PA, glycemic levels, and other health outcomes based on behavioral change techniques^9^ and the self‐determination theory.^10^ Wearable technology comprises devices that provide real‐time feedback on PA levels through means of step counts, the intensity of PA performed, or calories burned.^11^ They include activity trackers, pedometers, accelerometers, smartwatches, etc.^11^ Wearables are insightful in self‐management since immediate feedback is a behavior change tool to motivate users to maintain lifestyle changes.^12^, ^13^, ^14^ According to the self‐determination theory, participants would experience competence by seeing results from their efforts, feel a sense of affirmation and autonomy over their health through setting their own goals, and feel in control of their condition, thereby increasing intrinsic motivation to maintain lifestyle changes.^10^, ^15^ Through this, people with DM would be better able to monitor and manage their condition with minimal or no supervision in the long term. Consequently, adults with T2DM may increase PA, reduce glycemic levels, and improve other outcomes, such as blood pressure (BP), body mass index (BMI), and serum lipid profile after WT‐BPAI.

Nine existing systematic reviews (SRs) on the effectiveness of the WT‐BPAI on various health outcomes were found,^16^, ^17^, ^18^, ^19^, ^20^, ^21^, ^22^, ^23^, ^24^ with differing conclusions and limited by methodological limitations. Five had broad study populations such as chronic conditions, including chronic respiratory diseases, diabetes, cardiovascular diseases, cancer, and cognitive disorders, chronic respiratory diseases, diabetes^17^, ^22^ and cardiometabolic diseases,^19^, ^20^, ^24^ where results from these studies would be less significant in showing the true efficacy of WT‐BPAI when applied to adults with T2DM specifically.^25^ Luo et al.,^21^ Mattison et al.,^22^ Rodriguez‐Leon et al.,^23^ and Wang et al.^24^ included multiple categories of wearable‐based interventions using smartphone applications,^23^, ^24^ blood glucose measurement and medication administration devices,^21^ and intelligent foot insoles.^22^ With mixed interventions, assessing the efficacy of PA specifically would be difficult.

Five included studies with a mixture of study designs, such as a mixture of randomized controlled trials (RCTs) and quasi‐experimental studies,^16^, ^17^, ^24^ a mixture of RCTs, observational and feasibility studies,^22^ or a mixture of case–control and cohort studies.^23^ By including non‐RCTs, heterogeneity is increased,^25^ and conclusions drawn would be less convincing than SRs of RCTs which would provide the highest level of evidence.^26^ Rodriguez‐Leon et al.^23^ and Wang et al.^24^ did not search Embase, and all nine identified SRs did not search in technology‐specific databases, which could result in unidentified studies since Embase is considered an important database in biomedical research,^27^ and WT‐BPAI are based in the field of technology. Some were purely narrative,^18^, ^22^, ^23^, ^24^ where this would affect precision^28^ and generalizability of the results.^29^ Of those that had meta‐analyses, inconsistency was a problem.^16^, ^17^, ^19^, ^20^

Due to these limitations and ever‐changing advancements in technology, this SR aims to explore the effectiveness of WT‐BPAI, minimize knowledge gaps, and synthesize the best available evidence in DM management. Results from this review would inform practice by introducing a feasible intervention to increase PA uptake in adults with T2DM's self‐management routines, and potentially revolutionize future DM management. The review's objectives are to (1) evaluate the effectiveness of WT‐BPAI on PA levels, glycemic levels, and other outcomes (BP, BMI, and serum lipid profile) in adults with T2DM; and (2) investigate the potential covariates affecting aforementioned outcomes.

## 2 METHODS

This SR was conducted as per Cochrane's Handbook for SRs of Interventions,^30^ and the Preferred Reporting Items for Systematic Reviews and Meta‐Analyses (PRISMA) 2020 statement.^31^ The PRISMA checklist is presented in Table S1 and has been registered with the International Prospective Register of SRs (PROSPERO: CRD42023392742).

### 2.1 Eligibility criteria

The inclusion of studies followed the eligibility criteria (Table S2): Participants diagnosed with T2DM were 18 years of age or older, regardless of the severity of the condition or mode of treatment (oral or parenteral medication). The interventions incorporated wearable technology to specifically target the promotion of PA through wearable devices. The comparators in this study included usual care, waitlist, no treatment, usual care, use of the wearable device alone, coaching, or educational advice. Primary outcomes included PA and glycemic level outcomes, whereas secondary outcomes included BP, BMI, and serum lipid profile. We included only RCTs, without any date restrictions, and excluded studies with inaccessible full texts.

### 2.2 Information sources

We searched eight bibliographic databases, including PubMed, Embase, Cumulated Index to Nursing and Allied Health Literature, Cochrane, Web of Science, Scopus, Institute of Electrical and Electronics Engineers, and ProQuest Dissertation & Theses, for published and unpublished studies. We searched the Cochrane Central Registry of Controlled Trials and CinicalTrials.gov for potentially relevant trial documents. Existing similar SRs and relevant articles' reference lists were also hand‐searched. For studies with inaccessible full texts or missing information, we contacted the authors. Most authors did not respond, leading to insufficient information for an assessment of reporting bias.

### 2.3 Search strategy

Two concepts, “diabetes” and “wearable devices,” were combined using a three‐step search strategy. We identified initial key terms from PubMed and Embase by analyzing the titles, abstracts, and index terms of relevant articles. Concepts were then combined with the appropriate Boolean operators, and database‐specific searches were conducted for each database (Table S3). Validated RCT filters were utilized where available. The first reviewer (CR) refined the search strategy after consultation with a senior research librarian, with the final search strategy reviewed by the research team.

### 2.4 Selection process

The selection process was guided by the PRISMA statement^31^ and the Cochrane Handbook for SRs.^30^ All results were exported into Covidence software,^32^ duplicates were removed. Reviewer 1 (WWA) and reviewer 2 (YL) screened the titles and abstracts independently to exclude ineligible studies. We obtained the full texts of all potentially relevant studies. Two independent reviewers then pilot‐tested the eligibility criteria with eight articles and independently screened the full texts for inclusion. Disagreements between two reviewers (WWA and YL) that could not be resolved through discussion were resolved through consulting a third reviewer (STL).

### 2.5 Data extraction

We used a modified data extraction form, developed following the requirements of the Cochrane Handbook for SRs of Intervention,^33^ to obtain information on study characteristics, including authors, year of publication, country of study, setting, study design, population, mean age, sample size, interventions, wearable used in the intervention, model, comparators, outcomes, measures, attrition rates, use of intention‐to‐treat analysis (ITT) or missing data management (MDM), and grant. Intervention‐specific items include medium, materials, co‐interventions, duration, theoretical basis, reminder, feedback, and follow‐up. Piloting of the form and subsequent data extraction were done independently by between two reviewers (CR and AB) with any dispute resolved through discussion, or consultation with reviewer 3 (STL).

### 2.6 Quality appraisal of included studies

The quality assessment of included studies was independently evaluated by independent reviewers (WWA and YL) to minimize errors and provide different perspectives, using the Cochrane Collaboration's risk of bias tool version 2 (RoB 2).^34^ RoB 2 was assessed based on the randomization process, deviations from the intended intervention, missing outcome data, measurement of outcome, and selection of the reported result. Each domain was rated “yes,” “probably yes,” “probably no,” “no” or “no information.” The highest risk rating across all five domains determined the overall risk. To maximize reliability, WWA and YL piloted the RoB 2 on three articles to better understand the rating criteria and ensure consistency. We then assessed the domains and resolved any disputes through discussion or consultation with STL.

### 2.7 Interrater reliability

The study examined the inter‐rater reliability of two reviewers, WWA and YL, in the areas of trial selection, data extraction, trial quality rating, and overall evidence certainty using Cohen's Kappa (*κ*).^35^ The following ranges of values for *κ* were interpreted: 0.01–0.20, none to minor; 0.21–0.40, fair; 0.41–0.60, moderate; 0.61–0.80, significant; and 0.81–1.00, practically perfect agreement.^35^

### 2.8 Data synthesis

The R software version 4.3.1's package *meta* was applied to do the meta‐analysis, subgroup analysis, and meta‐regression studies.^36^ To get objective estimates, the limited maximum likelihood estimator for random‐effect meta‐analysis was used.^37^ The Hartung–Knapp adjustment involved estimating the between‐study variance and treating it as if it were known and fixed.^38^ This modified method justified using quantiles from a *t* statistic rather than a *Z* statistic for making inferences about the average effect.^38^ This method provided actual coverage probabilities, which was more accurate than the conventional method.^38^ To assess the genuine effects that might be anticipated for 95% of future similar trials, a 95% prediction interval (PI) was calculated.^39^ There was a statistically significant effect in the future if all 95% of values fall on the same side of the null.^39^ There was no appreciable impact in the future if all values fall inside both sides of the null.^39^ The continuous data were presented as mean difference (MD) or standardized MD (SMD) with standard deviation (SD) and 95% confidence intervals (CIs) using the inverse‐variance statistical approach.^40^, ^41^ A *t‐*test with *p* < 0.05 was used to assess the WT‐BPAI's overall impact.^36^

As most studies had small sample sizes, the Hedges' *g* was used to measure the effect size.^42^ The effect size was defined as very small (*g* = 0.01), small (*g* = 0.2), medium (*g* = 0.5), large (*g* = 0.8), and very large (*g* = 1.2).^43^, ^44^ Data transformation was done using formulas by Higgin et al. (2022).^45^ Intervention efficacy for each outcome was presented with forest plots. *I* ^*2*^ statistics was used to find heterogeneity.^40^ *I* ^*2*^ is the percentage of the observed variance that corresponds to the variance in true effects. The following criteria are used to interpret *I* ^*2*^ values: 0%–40% small, 30%–60% moderate, 50%–90% substantial, and 75%–100% significant.^40^

### 2.9 Additional data analysis

This review included subgroup and meta‐regression analyses to investigate the possibility of covariates impacting the treatment effect.^30^ For categorical covariates, subgroup analyses were conducted based on the wearable device type, the involvement of a goal‐setting component, and the intervention function. If *p* was less than 0.1, the subgroup effect was statistically significant.^46^ For continuous covariates, a series of univariate meta‐regression analyses were performed.^47^ A random‐effects meta‐regression model was used to investigate the influence of publication year, sample size, mean age, and duration of intervention. Regression coefficients (*β*) were the estimated change in the effect of the size units of the covariates on health outcomes.^48^ A significant effect was shown by a *p* < 0.5.^48^ We carried out a narrative synthesis if the data from selected trials were insufficient for meta‐analysis.

### 2.10 Certainty of evidence and publication biases

The overall quality of the evidence from the included studies was assessed using the Grading of Recommendations Assessment, Development and Evaluation (GRADE) criteria.^49^ The GRADE consists of five domains, including risk of biases, heterogeneity, indirectness of evidence, imprecision of results, and risk of publication bias.^49^ Certainty of evidence was graded as “high,” “moderate,” “low,” or “very low”^50^ using GRADEpro online software.^51^ Funnel plots and Egger's test were used to test for publication bias when 10 or more studies were included in a forest plot.^52^ With the aid of the funnel plot's asymmetry and an Egger test *p*‐value of less than 0.05, publication bias was determined.^52^

## 3 RESULTS

### 3.1 Study selection

**FIGURE 1:**
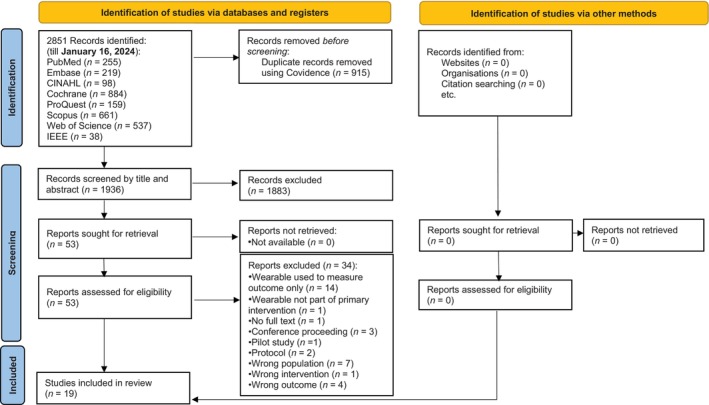
Preferred Reporting Items for Systematic Reviews and Meta‐Analyses (PRISMA) flow diagram of study selection. CINAHL, Cumulative Index of Nursing and Allied Health Literature, and IEEE, Institute of Electrical and Electronics Engineers Xplore digtial library.

A flow diagram of PRISMA (Figure 1) describes the search procedure. A search of eight electronic databases using Covidence^32^ produced 2851 records between the start date and January 16, 2024. By hand searching, no further records could be found. Covidence^32^ was used to eliminate 1881 duplicates, and YL and WWA screened 1936 entries based on their titles and abstracts. Thirty‐four studies were eliminated with reasons (Table S4) after the remaining records were evaluated against the qualifying criteria. Ultimately, this SR included 19 RCTs.^14^, ^53^, ^54^, ^55^, ^56^, ^57^, ^58^, ^59^, ^60^, ^61^, ^62^, ^63^, ^64^, ^65^, ^66^, ^67^, ^68^, ^69^, ^70^

### 3.2 Study characteristics and description of intervention

Table 1 summarizes the characteristics of the 19 RCTs involving 2547 participants from 14 countries including Australia (*n* = 1),^58^ Belgium (*n* = 1),^57^ Brazil (*n* = 1),^65^ Canada (*n* = 1),^70^ China (*n* = 1),^61^ India (*n* = 1),^68^ Japan (*n* = 2),^63^, ^64^ Mexico (*n* = 1),^53^ Netherlands (*n* = 1),^60^ Norway (*n* = 1),^56^ Oman (*n* = 1),^53^ Turkey (*n* = 1),^69^ the United Kingdom (*n* = 1),^54^ and the United States (*n* = 5).^14^, ^55^, ^62^, ^66^, ^67^ The trials were conducted from 2004^70^ to 2022,^63^ with a mean age range of 44.2^53^ to 68.7^65^ years. Sample sizes ranged from 30^55^, ^59^ to 593.^54^ Of the 19 RCTs, five trials^53^, ^60^, ^63^, ^69^, ^70^ adopted a theoretical basis for developing their interventions. The types of wearables used included pedometers (*n* = 11), accelerometers (*n* = 1),^63^ smartwatches (*n* = 3),^60^, ^62^, ^69^ a combination of pedometers and accelerometers (*n* = 1),^59^ activity trackers (*n* = 2),^64^, ^66^ and a heart rate monitor (*n* = 1).^61^ The intervention duration ranged from 5 weeks^59^ to 1 year.^53^, ^54^, ^66^, ^67^ All interventions included a monitoring function, and three of them included a reminder function.^62^, ^66^, ^69^ Intervention details are reported in Table S5.

**TABLE 1:** Characteristics of included 19 RCTs in adults with type 2 diabetes mellitus.

Author (year)/country	Settings	Design	Age (mean)	Sample size	Intervention	Wearable used in intervention (I) (model)/ Functions	Comparator (C)	Outcomes (measure)	Attrition rate (%)	ITT/MDM	Grant
Alghafri et al. (2018)/Oman	Community + Hospital (Multicentre)	2‐arm cluster RCT	44.20	T: 232 I: 122 C: 110	‘MOVEdiabetes’ intervention	Pedometer (Amax DigiWalker SW‐200)/monitoring	Usual care	Glycemic level (HbA1c) Physical activity (step/day) Others (BMI, HDL, LDL, SBP, DBP)	25	Y/Y	Y
Andrews et al. (2011)/UK	Community + Hospital (Multicentre)	3‐arm RCT	59.96	T: 593 I_1_: 248 I_2_: 246C: 99	I_1_: intensive dietary support I_2_: intensive dietary support and activity	Pedometer (Yamax Digiwalker CW200)/monitoring	Usual care	Glycemic level (HbA1c) Physical activity (MVPA) Others (BMI, HDL, LDL, SBP, DBP)	2.36	Y/N	Y
Araiza et al. (2006)/US	Hospital	2‐arm RCT	50.00	T: 30 I: 15 C: 15	Pedometer‐based physical activity program	Pedometer (Yamax Digiwalker SW‐701)/monitoring	Pedometer + Normal baseline activity	Glycemic level (HbA1c, FBG) Physical activity (step/day) Others (BMI, HDL, LDL, SBP, DBP)	0	N/N	Y
Bjørgaas et al. (2008)/Norway	Hospital	2‐arm RCT	58.90	T: 48 I: 23 C: 25	Pedometer‐based physical activity program	Pedometer (Yamax DigiWalker ML AW‐320)/monitoring	Goal setting	Glycemic level (HbA1c, FBG) Others (HDL, SBP, DBP)	31.40	N/Y	Y
Diedrich et al. (2010)/US	Hospital	2‐arm RCT	55.81	T: 53 I: 27 C: 26	Diabetes Self‐Management Education Program	Pedometer (Yamax Digiwalker SW‐200)/monitoring	DSMEP (diabetes self‐management education program)	Glycemic level (HbA1c) Others (SBP, DBP)	38	N/N	Y
Engel & Lindner (2006)/Australia	Community	2‐arm RCT	62.00	T: 54 I: 24 C: 30	Pedometer and coach	Pedometer (Yamax DigiWalker‐700) /monitoring	Coaching	Glycemic level (HbA1c) Others (BMI, SBP, DBP)	12	N/N	Y
Greef et al. (2011)/Belgium	Hospital	2‐arm RCT	62.00	T: 92 I: 60 C: 32	Pedometer‐based behavioural modification program with telephone support	Pedometer /monitoring	Pedometer + usual care	Physical activity (Step/day, MVPA)	4.35	Y/N	N
Kempny et al. (2008)/Poland	Hospital	2‐arm RCT	57.00	T: 30 I: 16 C: 14	Pedometer	Pedometer/accelerometer (Omron HJ113) /monitoring	Oral advice to increase PA	Physical activity (IPAQ)	NR	N/N	N
Kooiman et al. (2018)/Netherlands	Hospital	2‐arm RCT	56.00	T: 72 I: 40 C: 32	Online self‐tracking (eHealth) program	Fitbit Zip /monitoring	Usual care	Glycemic level (HbA1c) Others (BMI)	8.33	Y/Y	Y
Li et al. (2021)/China	Community + Hospital (Multicentre)	2‐arm RCT	48.20	T: 101 I: 55 C: 46	mHealth App and chest‐wearable remote exercise monitoring intervention	HR band (Recovery Plus Inc)/monitoring	Unsupervised prescribed exercise	Glycemic level (HbA1c) Others (BMI)	15.80	N/N	Y
Lystrup et al. (2020)/USA	Hospital	2‐arm RCT	63.50	T: 120 I: 60 C: 60	Fibit + friends	Fitbit charge/monitoring + reminder	Fitbit [soloFitbit group]	Glycemic level (HbA1c) Physical activity (step/day)	12	N/N	Y
Matsushita et al. (2022)/Japan	Hospital	2‐arm RCT	61.65	T: 36 I: 18 C: 18	Combination of exercise instruction with ambulatory accelerometer	Accelerometer (active style Pro HJA‐750C)/monitoring	Accelerometer	Glycemic level (HbA1c, FBG) Physical activity (step/day) Others (BMI, HDL, SBP, DBP)	19.40	N / N	Y
Miyauchi et al. (2016)/Japan	Hospital	2‐arm RCT	62.30	T: 200 I: 100 C: 100	Activity monitory	Activity monitor (MT‐KT01, terumo)/monitoring	Pedometer + exercise target	Glycemic level (HbA1c)	6.50	N/N	Y
Oliveira et al. (2022)/Brazil	Community + Hospital	2‐arm RCT	68.70	T: 44 I: 22 C: 22	Receiving guidance on a DASH‐type diet and increased physical activity with the help of a pedometer for step counting	Pedometer (HJ‐321, Omron®)/monitoring	Dietary guidance [DASH]	Glycemic level (HbA1c, FBG) Physical activity (step/day) Others (BMI, HDL, SBP, DBP)	20.45	N/N	Y
Patel et al. (2021)/US	Hospital	4‐arm RCT	52.50	T: 361 I1: 92 I2: 95 I3: 87 C: 87	I_1_: gamification with support I_2_: Gamification with collaboration I_3_: gamification with competition	Activity tracker (withings activite steel)/monitoring + reminder	Activity tracker + feedback from device (regular feedback and goal setting from the devices and smartphone application)	Glycemic level (HbA1c, FBG) Physical activity (step/day) Others (LDL)	6.93	Y/Y	Y
Piette et al. (2011)/US	Hospital	2‐arm RCT	56 .00	T: 291 I: 145 C: 146	Telephone‐delivered cognitive‐behavioral therapy program	Pedometer (Omron HJ‐720 ITC)/monitoring	Enhanced usual care	Glycemic level (HbA1c) Physical activity (step/day) Others (SBP, DBP)	14	Y/N	Y
Shenoy et al. (2010)/India	Hospital	2‐arm RCT	52.08	T: 40 I: 20 C: 20	Aerobic walking program using heart rate monitor and pedometer	Pedometers /monitoring	Usual care	Glycemic level (HbA1c, FBG) Others (BMI, SBP, DBP)	NR	N/N	N
Timurtas et al. (2022)/Turkey	Hospital	3‐arm RCT	51.60	T: 90 I1: 30 I2: 30 C: 30	I_1_: Mobile app I_2_: Smartwatch	Smartwatch (DIABETEX) /monitoring + reminder	Supervised prescribed individually‐tailored exercise training	Glycemic level (HbA1c)	6.67	Y/Y	Y
Tudor‐locke et al. (2004)/Canada	Hospital	2‐arm RCT	52.90	T: 60 I: 30 C: 30	First step program	Pedometer (Yamax SW‐200)/monitoring	Waitlist control	Glycemic level (HbA1c) Physical activity (Step/day) Others (HDL, LDL, SBP, DBP)	36.70	N/N	Y

Abbreviations: BMI, body mass index; C, control group; DASH, Dietary Approaches to Stop Hypertension; DBP, diastolic blood pressure; DM, diabetes mellitus; FBG, fasting blood glucose; HbA1c, glycated hemoglobin; HDL: high‐density lipoprotein; I, intervention group; IPAQ, the International Physical Activity Questionnaire; ITT, intention‐to‐treat analysis; LDL, low‐density lipoprotein; MDM, missing data management; MVPA, moderate to vigorous activity; N, no; RCT, randomized control trial; SBP, systolic blood pressure; T, total; Y, yes.

### 3.3 Risk of bias in studies

The risk of bias summary according to the RoB 2 criterion is displayed in Figure S1. A Cohen's kappa score of 0.89, which indicates nearly perfect agreement, was obtained from two reviewers.^35^ Twelve RCTs used per‐protocol analysis, and seven ITT studies were included in the total of 19 RCTs. Seventy trials (89.5%) did not provide information on allocation concealment. As all participants knew their allocated intervention during the trial, there were some concerns regarding deviations from the intended intervention. A total of 10 trials (52.6%) did not perform to register in clinical trial registries or publish a protocol. As a result, there were reservations regarding the overall risk of bias in all the included trials. The attrition rates ranged from 0%^55^ to 36.70%.^70^ An attrition rate of less than 5% was seen in just three studies. Eight of them used either ITT or MDM.

### 3.4 Glycemic outcomes

**FIGURE 2:**
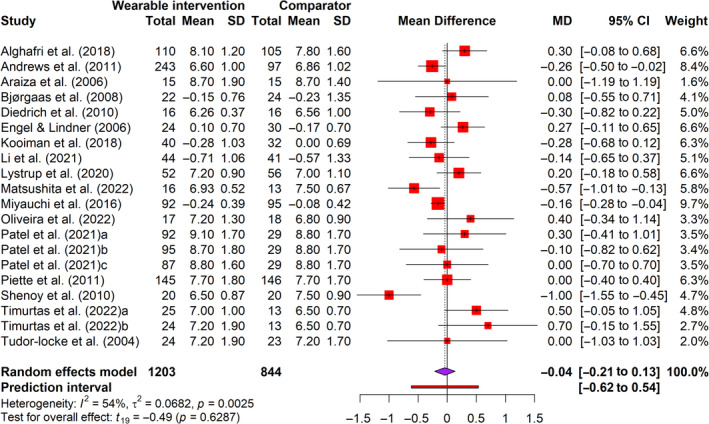
Forest plot of mean difference in glycated hemoglobin (HbA1c) for wearable technology‐based physical activity interventions and comparators. CI, confidence interval; MD, mean difference; SD, standard deviation.

Seventeen trials^14^, ^53^, ^54^, ^55^, ^56^, ^58^, ^60^, ^61^, ^62^, ^63^, ^64^, ^65^, ^66^, ^67^, ^68^, ^69^, ^70^ in 20 arms involving 2407 participants reported glycated hemoglobin (HbA1c) levels and six trials^55^, ^56^, ^63^, ^65^, ^68^, ^70^ involving 224 participants reported fasting blood glucose (FBG) as the glycemic outcome. The HbA1c levels measure glycemia over 90 days,^71^ and FBG readings measure glycemia at the point of care.^72^ Figure 2 showed that interventions did not lead to a significant change in HbA1c (*t* = 0.49, *p* = 0.63), where HbA1c decreased by 0.04% (95% CI = −0.21 to 0.13) with a very small effect size (*g =* −0.05) (Figure S2) when compared with the comparator. Similarly, interventions did not lead to a significant change in FBG (*t* = −0.77, *p* = 0.48), with a small effect size (*g* = −0.34, 95% CI = −1.50 to 0.81) (Figure S3). Moderate and substantial heterogeneities were detected (*I* ^2^ = 54%–86%).

### 3.5 Physical activity

**FIGURE 3:**
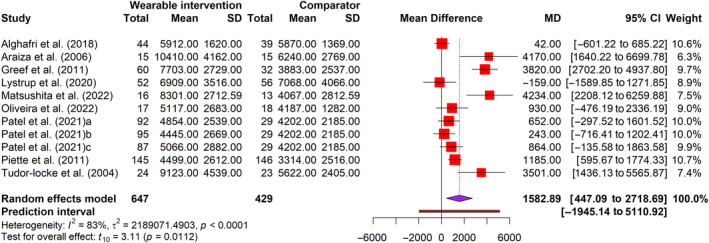
Forest plot of mean difference in step count (steps per day) for wearable technology‐based physical activity interventions and comparators. CI, confidence interval; MD, mean difference; SD, standard deviation.

We measured the outcomes of PA in this review in step count (steps per day) and moderate‐to‐vigorous physical exercise (MVPA) using pedometers or accelerometers. (Table 2). We found 11 arms of 9 RCTs^53^, ^55^, ^56^, ^62^, ^63^, ^65^, ^66^, ^67^, ^70^ with 1076 participants for steps per day (Figure 3). Meta‐analysis revealed a significant (*t* = 3.11, *p* < 0.05) increase of 1583 steps/day (95% CI = 477–2719), with medium effect size (*g =* 0.54) favoring intervention (Figure S4). The PI ranged from −0.56 to 1.64, with values found on either side of the null, suggesting that the intervention did not substantially decrease the number of steps taken daily when compared to a comparator in comparable future research. However, substantial heterogeneity was detected (*I* ^*2*^ = 83%). Only two trials^54^, ^57^ involving 432 participants were found for MVPA. Meta‐analysis reveals that interventions resulted in a nonsignificant increase (*t* = 3.10, *p* = 0.20) in MVPA by 7.69 min per day (95% CI = −23.83 to 39.20), with a small effect size (*g* = 0.34). (Figure S5) Unimportant heterogeneity was observed (*I* ^*2*^ = 29%).

**TABLE 2:** Meta‐analysis of wearable technology‐based physical activity interventions on all outcomes.

Outcomes	N	Sample size	Mean difference (95% CI)	Prediction interval	*t‐*value	*P*‐value	*I* ^*2*^ (%)	Hedges’ *g* (95% CI)	Prediction interval	*t‐*value	*P‐*value	*I* ^*2*^ (%)
Glycemic level												
HbA1c	20	2047	−0.04 (−0.21, 0.13)	−0.62, 0.54	−0.49	0.63	54%	−0.05 (−0.22, 0.12)	−0.53, 0.43	−0.62	0.54	53%
FBG	6	224	−7.60 (−27.41, 12.21)	−61.56, 46.35	−0.99	0.37	93%	−0.34 (−1.50, 0.81)	−3.42, 2.74	−0.77	0.48	86%
Physical activity												
Step/day	11	1076	1583 (447, 2719)	−1945, 5111	3.11	<0.05*	83%	0.54 (0.18, 0.90)	−0.56, 1.64	3.31	<0.01**	76%
MVPA	2	432	7.69 (−23.82, 39.20)	NA	3.10	0.20	11%	0.34 (−1.41, 2.09)	NA	2.48	0.24	29%
Others												
BMI	9	900	−0.25 (−0.58, 0.08)	−0.71, 0.20	−1.74	0.12	9%	−0.18 (−0.32, −0.03)	−0.47, 0.12	−2.83	0.02*	0%
HDL	7	740	0.02 (−0.04, 0.07)	−0.11, 0.15	0.82	0.44	0%	0.05 (−0.08, 0.19)	−0.15, 0.25	0.94	0.38	0%
LDL	7	993	−0.06 (−0.16, 0.05)	−0.24, 0.13	−1.30	0.24	0%	−0.05 (−0.15, 0.05)	−0.23, 0.13	−1.31	0.24	0%
SBP	11	1158	−2.46 (−4.13, −0.79)	−4.59, −0.33	−3.28	<0.01**	0%	−0.15 (−0.30, < 0.001)	−0.35, 0.05	−2.18	0.05	16%
DBP	11	1158	−1.00 (−3.95, 1.96)	−9.29, 7.30	−0.75	0.47	75%	−0.13 (−0.52, 0.26)	−1.24, 0.98	−0.74	0.48	70%

Abbreviations: BMI, body mass index; CI, confidence Interval; DBP, diastolic blood pressure; FBG, fasting blood glucose; HbA1c, glycated hemoglobin; HDL: high‐density lipoprotein; Hedges' *g*, effect size; LDL: low‐density lipoprotein; MVPA, moderate to vigorous activity; N, number of arms; NA, not applicable; SBP, systolic blood pressure; *t*‐value, T statistics.

* *p* < 0.5;

** *p* < 0.1.

### 3.6 BP, BMI, and serum lipid profile

Other outcomes included BP involving 1158 participants from 11 RCTs,^14^, ^53^, ^54^, ^55^, ^56^, ^58^, ^63^, ^65^, ^67^, ^68^, ^70^ BMI involving 900 individuals from 9 RCTs,^53^, ^54^, ^55^, ^58^, ^60^, ^61^, ^63^, ^65^, ^68^ high‐density lipoprotein (HDL) involving 740 participants from 7 RCTs,^53^, ^54^, ^55^, ^56^, ^63^, ^65^, ^70^ and low‐density lipoprotein (LDL) involving 993 participants from seven arms of five RCTs.^53^, ^54^, ^55^, ^66^, ^70^ For BP, meta‐analyses showed that intervention resulted in a significant decrease (*t* = −3.28, *p* < 0.01) in systolic BP (SBP) by 2.46 mm Hg (95% CI = −4.13 to −0.79) but not a significant decrease (*t* = −0.75, *p* = 0.470) in diastolic BP (DBP) by 1.00 mm Hg (95% CI = −3.95 to 1.96) when compared with the comparator (Figures S6 and S7). The effect sizes for SBP and DBP were very small effect size (*g* = −0.15 to −0.13). The PI (95%) was discovered on both sides of the null, suggesting that the intervention did not substantially decrease the SBP when compared with a comparator in similar studies in the future. No heterogeneity (*I* ^2^ = 0%) was observed for SBP, whereas substantial heterogeneity (*I* ^2^ = 75%) was detected for DBP. There were no significant differences between the intervention and control groups on the MDs of the BMI, HDL, or LDL (Figures S8–S10). However, Figure S8 shows that the intervention significantly reduced the effect size of BMI (*t* = −2.83, *p* = 0.02) with a very small effect size (*g* = −0.18) when compared to the comparator. No heterogeneities (*I* ^2^ = 0%–9%) were detected.

### 3.7 Subgroup and meta‐regression analyses

We ran a series of subgroup analyses and meta‐regression analyses for dichotomous and continuous covariates, respectively, where the results came from ten or more RCTs. Table 3 illustrates the results of subgroup analyses of wearable types, goal setting, and function on HbA1c, steps per day, SBP, and DBP (Figures S11–S20). The intervention for the sole monitoring function showed a significantly larger effect size (*p* = 0.02) on HbA1c reduction (*g* = −0.14, 95% CI: −0.36 to 0.08) compared with the intervention for the monitoring and reminder function (*g* = 0.15, 95% CI: −0.06 to 0.35). The effect sizes on increasing the number of steps taken each day were significantly larger (*p* < 0.01) when the intervention was only for monitoring function (*g* = 0.80, 95% CI: 0.28–1.31) compared with when it was both for monitoring and reminder function (*g* = 0.15, 95% CI: −0.12 to 0.41). There were no significant subgroup differences between the wearable device used, and involvement in goal setting on steps per day, HbA1c, SBP, and DBP.

**TABLE 3:** Subgroup analyses of wearable technology‐based physical activity interventions on categorical outcomes.

Outcomes	Categorical covariates	Subgroups	No. of arms	Sample size	Heterogeneities (*I* ^*2*^)	Hedges’*g* [ 95% CI]	Subgroup difference *p*‐value
Hemoglobin A1C (HbA1c)	Wearable device	Pedometer	10	1130	58%	−0.05 [−0.32, 0.23]	0.97
		Not a pedometer	10	917	53%	−0.05 [−0.31, 0.20]	
	Goal setting	Goal setting component	17	1771	48%	−0.04 [−0.21, 0.13]	0.46
		No goal setting component	3	276	78%	−0.29 [−1.74, 1.16]	
	Function	Monitoring only	14	1503	59%	−0.14 [−0.36, 0.08]	0.02*
		Monitoring + Reminder	6	544	0%	0.15 [−0.06, 0.35]	
Steps per day	Wearable device	Pedometer	6	578	78%	0.71 [0.16, 1.26]	0.20
		Not a pedometer	5	498	64%	0.31 [−0.32, 0.94]	
	Goal setting	Goal setting component	9	964	75%	0.52 [0.14, 0.90]	0.80
		No goal setting component	2	112	89%	0.71 [−8.75, 10.00]	
	Function	Monitoring only	7	607	78%	0.80 [0.28, 1.31]	< 0.01**
		Monitoring + Reminder	4	469	0%	0.15 [−0.12, 0.41]	
Systolic blood pressure	Wearable device	Pedometer	10	1129	22%	−0.16 [−0.32, 0.01]	0.53
		Not a pedometer	1	29	NA	0.08 [−0.65, 0.81]	
	Goal setting	Goal setting component	8	883	20%	−0.11 [−0.30 0.07]	0.34
		No goal setting component	3	275	0%	−0.25 [−0.78, 0.28]	
Diastolic blood pressure	Wearable device	Pedometer	10	1129	70%	−0.08 [−0.50, 0.33]	0.15
		Not a pedometer	1	29	NA	−0.70 [−1.46, 0.06]	
	Goal setting	Goal setting component	8	883	72%	−0.04 [−0.55, 0.47]	0.36
		No goal setting component	3	275	77%	−0.38 [−1.72, 0.95]	

* *p* < 0.5;

** *p* < 0.1.

Table 4 shows the results of meta‐regression analyses to explore whether the year of publication, sample size, mean age, and duration of intervention influence intervention effects. Results revealed that the intervention duration and participants' mean age were significant covariates for the steps per day (*β* = −0.08, *p* = 0.03) and SBP (*β* = 0.02, *p* = 0.03), respectively. The findings revealed that the shorter duration intervention significantly increased daily steps in comparison to the longer duration intervention. The result also indicated that younger adults significantly decreased their SBP after interventions compared to older adults. However, the year of publication and sample size did not influence steps per day, HbA1c, SBP, and DBP.

**TABLE 4:** Random‐effects meta‐regression analyses of wearable technology‐based physical activity interventions on continuous outcomes.

Outcomes	Continuous covariates	β	SE	95% lower	95% upper	*t‐*Value	*p*‐Value
Hemoglobin A1C (HbA1c)	Year of publication	0.01	0.01	−0.02	0.04	0.75	0.46
	Sample size	<−0.01	<0.01	<−0.01	<0.01	−0.53	0.60
	Mean age	<−0.01	0.01	−0.04	0.02	−0.54	0.60
	Duration of intervention	0.02	0.02	−0.02	0.06	0.93	0.36
Steps per day	Year of publication	−0.04	0.02	−0.10	<0.10	−1.92	0.09
	Sample size	<−0.01	<0.01	<−0.01	<0.01	−1.65	0.13
	Mean age	0.02	0.03	−0.04	0.08	0.78	0.46
	Duration of intervention	−0.08	0.03	−0.15	<−0.01	−2.57	0.03*
Systolic blood pressure	Year of publication	−0.01	0.02	−0.05	0.02	−0.69	0.51
	Sample size	<0.01	<0.01	<−0.01	<0.01	−0.02	0.98
	Mean age	0.02	<0.01	<0.01	0.04	2.59	0.03*
	Duration of intervention	<−0.01	0.02	−0.05	0.03	−0.46	0.65
Diastolic blood pressure	Year of publication	<−0.01	0.03	−0.08	0.06	−0.28	0.78
	Sample size	<0.01	<0.01	<−0.01	<0.01	0.42	0.69
	Mean age	<0.01	0.03	−0.06	0.07	0.32	0.75
	Duration of intervention	0.04	0.04	−0.05	0.14	0.98	0.35

Abbreviations: *β*, regression coefficients; SE, standard error; *t*‐value, T statistics.

* *p*‐Value <0.5.

### 3.8 Narrative synthesis

Among 19 RCTs, one study^59^ did not provide sufficient data for steps per day, BP, glucose level, and serum lipid profile after intervention in the report. Our team emailed the authors, requesting supplementary data, but there was no response after three reminders. Hence, narrative analysis was done. Participants in the WT‐BPAI group achieved a significantly higher level of PA after 1 and 5 weeks than those in the control group. There were no changes in BP, BMI, glycemic level, and serum lipid profile. These patterns of findings resembled the results of the meta‐analysis.

### 3.9 Certainty of evidence and publication bias

Following the GRADE criteria, all outcomes had very low levels of evidential confidence (Table S6). The randomization process, deviations from the intended intervention, and missing outcome data all contributed to the downgraded RoB. High heterogeneity of outcomes downgraded inconsistency. The different types of wearables led to a downgrade in the domain of indirectness. The small sample size and wide confidence intervals have induced a downgrade in the domain of imprecision. Publication bias was assessed for steps per hour, HbA1c, SBP, DBP, and BMI, as more than 10 studies were included in their meta‐analyses Based on Egger's test and visual inspection of funnel plots, no publication bias was detected for step count (*p* = 0.20), HbA1c (*p* = 0.93), SBP (*p* = 0.59), and DBP (*p* = 0.48) (Figures S21–S24).

## 4 DISCUSSION

### 4.1 Summary of findings

This review included a total of 19 trials involving 2574 adults with T2DM across 14 countries. Meta‐analyses identified that the WT‐BPAI significantly increased steps per day and SBP when compared with the comparator group. Subgroup analyses revealed that interventions with monitoring functions had a significantly greater effect on lowering HbA1c and increasing the number of steps taken each day than interventions with monitoring and reminder functions. Random‐effects meta‐regression revealed that the shorter the length of the intervention and the younger the person were found to be significant covariates for increasing the number of steps taken each day and lowering SBP, respectively. Based on RoB 2, there were some concerns in over half of the studies because of the randomization process, deviations from the intended intervention, and missing outcome data. Publication bias was not detected. According to the GRADE criteria, there was very low overall certainty in the evidence.

### 4.2 Glycemic outcomes

Our meta‐analyses showed that the WT‐BPAI reduced HbA1c and FBG when compared with the comparators, but the differences were not statistically significant. This is like findings from similar SRs^16^, ^17^, ^18^ that reported no significant effect on glycemic levels. Intervention may help to increase PA by increasing muscular contractions to promote glucose uptake,^73^ resulting in decreased glycemic levels.^74^ However, solely PA may not significantly reduce glycemic levels. Notably, one study has attrition rates as high as 36.70%.^70^ The WT‐BPAI may have become less novel and challenging over time, making it harder for users to track their PA and achieve their health objectives. As a result, it may have become more important for users to stay motivated to engage in PA beyond simply understanding their PA levels.^10^ Hence, the lack of statistical significance is probably due to the individuals' struggles to maintain consistent PA as the intervention advanced, rather than the ineffectiveness of interventions in lowering glycemic levels. Furthermore, adults with T2DM were administered different medications to control glycemic levels according to their situations.^16^ That could explain the lack of effect on HbA1c and FBG.

### 4.3 Physical activity

Based on meta‐analyses, WT‐BPAI significantly increased PA level with a medium effect size for step count, where step count increased by around 1583 steps/day. This is similar to findings by Baskerville et al.,^16^ Franssen et al.,^17^ Hodkinson et al.,^19^ Kirk et al.,^20^ and Wang et al.^24^ The possible explanation is that people with diabetes were aware of PA levels through immediate quantitative feedback from wearables,^75^, ^76^ where this awareness promotes self‐evaluation and self‐reinforcement of the behavior change.^77^ It may explain the increased steps per day after the intervention. However, we cannot find significant differences between intervention and comparator on MVPA. The nonsignificant results may be due to the limited sample size of the selected two trials in a meta‐analysis. A small sample size would have insufficient statistical power to differentiate the differences between the two groups.^78^ Therefore, we should interpret MVPA findings cautiously. In line with a previous review,^16^ the effect sizes from the accelerometer and pedometer were comparable in our subgroup analyses. There was no significant difference in all outcomes between wearable types. Given that pedometers are a cost‐effective means to monitor the steps per day, future studies can consider pedometers as wearable devices for monitoring physical activities. Our meta‐regression showed that the short duration had a better effect on increasing steps per day when compared with a long duration of intervention. The findings align with a prior SR^21^ that the wearable device‐based intervention with a shorter duration had a better effect. This might account for a drop in motivation to participate and adherence over an extended period.^21^

### 4.4 Blood pressure

The intervention had statistical significance in reducing SBP, but not DBP. These results were like a recent SR^17^ on a significantly decreased SBP after wearable activity tracker‐based interventions. These findings can be explained by PA may maintain healthy BP through the body's adaptive mechanisms involving blood circulation and metabolic changes.^79^ Given that a difference in statistical significance was observed between SBP and DBP reduction, the intervention can reduce SBP more effectively than DBP.^80^, ^81^ There is a correlation between a decrease in SBP and a decrease in stroke mortality.^82^ Hence, the WT‐BPAI may lower the risk of cardiovascular disease.^17^

### 4.5 Body mass index

Our meta‐analyses revealed that adults in the WT‐BPAI group did not decrease the MD in BMI, but significantly reduced the effect size of BMI when compared with the comparator group. Considering that a high level of sedentary behavior was found in adults with T2DM,^83^ WT‐BPAI might act as a motivational tool by increasing the motivation and encouragement of physical activities and translating the goals into practice.^84^ Physical activities could help adults in the WT‐BPAI increase their energy expenditures.^16^ Hence, the WT‐BPAI has an indirect effect by increasing weight loss, which could lower the effect size of BMI.

### 4.6 Serum lipid profile

Our SR revealed that there are no appreciable variations in LDL or HDL levels between the WT‐BPAI and comparator and groups. The lipid profile showed no significant differences, which is like a previous SR^16^ of the impact of accelerometer and pedometer use in people with T2DM. Notably, another SR^17^ found that wearable activity tracker‐based interventions showed significantly decreased LDL among individuals with any chronic disease. The different populations may be the cause of these discrepancies. Comparable to glycemic results, varying individuals may be taking various drugs to regulate their lipid profiles, which might partially account for the absence of impacts on HDL and LDL.^16^ It is necessary to do more research on the WT‐BPAI across different types of populations.

### 4.7 Function and duration of intervention

Our subgroup analysis observed that the WT‐BPAI with just the monitoring function had a greater effect on lowering HbA1c and increasing daily steps than the WT‐BPAI with both the monitoring and reminder functions. The reminder function might not promote engagement in the intervention because participants had difficulties understanding the messages and notifications, which caused them to ignore the reminders.^85^ However, only three trials^62^, ^66^, ^69^ included both monitoring and reminder features; further study was required to validate the outcome. We observed significantly larger effect sizes when the intervention was in younger adults on SBP reduction than older adults in subgroup analyses. One possibility is that young adults regularly engage in PA to improve BP because they are familiar with wearable functions.^86^, ^87^ Another possible explanation for this finding is that older adults may not adhere to the use of wearable technology.^88^ As a result, the WT‐BPAI has the potential to lower SBP more effectively in young adults than in old adults.

### 4.8 Strengths and limitations

There are various advantages to our review. This review was able to minimize bias and overcome the methodological limitations of past comparable reviews by thorough, rigorous, and systematic searches and analyses.^27^ The present review identified a greater number of RCTs and no evidence of publication bias. Subgroup and meta‐regression analyses were employed to investigate the potential covariates affecting PA levels, glycemic levels, BP, BMI, and serum lipid profile in this study. We used objective outcomes in our review to prevent social desirability bias. To avoid paradoxical effects, a random‐effects meta‐analysis was conducted with the Hartung–Knapp adjustment.^38^ Furthermore, the 95% PI of the meta‐analyses was supplied to predict the outcome in future situations.^39^

However, there are some limitations. First, there were substantial heterogeneities created by many inter‐study differences, such as the variety of co‐interventions, length of intervention, staff participation, and comparators, which might have an impact on the actual outcomes in different trials. Hence, the total effect may have been incorrectly calculated due to the significant heterogeneity of several outcomes. Second, the fact that only English‐language research was included might restrict how far the results can be applied. Third, there was a lack of information from the selected trials about how active participants were and mortality severity at baseline, which may have affected their PA levels. Fourth, there is uncertainty regarding the intervention's durability because all outcomes were utilized after the intervention. Finally, the rating of the overall evidence as very low quality raises the possibility of eliminating the internal validity of the findings.

### 4.9 Implications to clinical practice

Because the overall evidence of all outcomes was very low in this review, we provided limited information for clinical decision‐making. However, the WT‐BPAI can consider supplementing interventions to increase daily steps and reduce SBP in addition to routine diabetic care. Based on our subgroup and meta‐regression results, the findings can provide direction for future WT‐BPAI design. The short duration of the WT‐BPAI is preferable, and the function of the intervention is solely for monitoring, which could be an effective and workable way to boost PA. The target population for the WT‐BPAI could benefit young adults with T2DM by decreasing their SBP. This would transform DM self‐management in the future by empowering patients to take better care of and control their health via wearable technology.

### 4.10 Future research

First, future trials could investigate the use of a short‐duration WT‐BPAI with only a monitoring function for young adults with T2DM. This may increase the efficacy of therapies in encouraging adherence and allow for a long‐term evaluation of their impact on glucose levels in subsequent reviews. Second, trials should consider making the availability of their protocols to prevent unnecessary ambiguity in reporting bias. Third, the very low overall quality of evidence in our review reminds us that future trials should provide information for allocation concealment and design a way for blinding participants. Lastly, as PA intervention mainly affects lean mass rather than fat mass,^89^ both lean and fat mass must be included in the outcomes. Nevertheless, only three trials^14^, ^55^, ^61^ reported body fat (%) and two trials^63^, ^65^ reported body fat mass (kg) in this review. A study showed that increased glycemic levels were hindered by low skeletal muscle mass and poor skeletal muscle tissue quality.^90^ Given that skeletal muscular mass plays a role in T2DM,^90^ further investigations are needed.

## 5 CONCLUSION

As the prevalence of diabetes increases globally, both the healthcare and economic sectors are being increasingly affected, and the need for an effective and feasible method to promote PA in adults with T2DM continues to rise. Through a comprehensive systematic search pooling of results from 19 RCTs, results suggest that WT‐BPAI can significantly improve steps per day and SBP. Future research should be conducted in a well‐designed trial with long‐term follow‐up outcomes to finally achieve a truly effective and sustainable intervention to increase PA in adults with T2DM.

## AUTHOR CONTRIBUTIONS

RECC, STL, and YL conceptualized and designed the study. RECC and AAYFB selected, extracted, and appraised articles. RECC and WWA carried out data analysis and synthesis of data. RECC wrote the manuscript, and YL reviewed the manuscript. All authors reviewed critically, read, and approved the final version of the article.

## FUNDING INFORMATION

Nil.

## CONFLICT OF INTEREST STATEMENT

The authors declare no conflicts of interest.

## Supporting information


**Data S1.** Supporting information.
