# Supplementary material for: Effectiveness of wearable technology‐based physical activity interventions for adults with type 2 diabetes mellitus: A systematic review and meta‐regression
Source: J Diabetes. 2024 Oct 4;16(10):e70002. doi: 10.1111/1753-0407.70002 (PMC11450597; doi:10.1111/1753-0407.70002)
Supplement: Supplementary file 1 — Data S1. Supporting information. [file JDB-16-e70002-s001.docx]

# SUPPLEMENTARY TABLE S1. PRISMA CHECKLIST

| **Section and Topic** | **Item #** | **Checklist item** | **Location where item is reported** |
| --- | --- | --- | --- |
| **TITLE** | | |  |
| Title | 1 | Identify the report as a systematic review. | Title page |
| **ABSTRACT** | | |  |
| Abstract | 2 | See the PRISMA 2020 for Abstracts checklist. | 3 |
| **INTRODUCTION** | | |  |
| Rationale | 3 | Describe the rationale for the review in the context of existing knowledge. | 5-7 |
| Objectives | 4 | Provide an explicit statement of the objective(s) or question(s) the review addresses. | 7 |
| **METHODS** | | |  |
| Eligibility criteria | 5 | Specify the inclusion and exclusion criteria for the review and how studies were grouped for the syntheses. | 7 |
| Information sources | 6 | Specify all databases, registers, websites, organisations, reference lists and other sources searched or consulted to identify studies. Specify the date when each source was last searched or consulted. | 8 |
| Search strategy | 7 | Present the full search strategies for all databases, registers and websites, including any filters and limits used. | 8 |
| Selection process | 8 | Specify the methods used to decide whether a study met the inclusion criteria of the review, including how many reviewers screened each record and each report retrieved, whether they worked independently, and if applicable, details of automation tools used in the process. | 8 |
| Data collection process | 9 | Specify the methods used to collect data from reports, including how many reviewers collected data from each report, whether they worked independently, any processes for obtaining or confirming data from study investigators, and if applicable, details of automation tools used in the process. | 9 |
| Data items | 10a | List and define all outcomes for which data were sought. Specify whether all results that were compatible with each outcome domain in each study were sought (e.g. for all measures, time points, analyses), and if not, the methods used to decide which results to collect. | 9 |
|  | 10b | List and define all other variables for which data were sought (e.g. participant and intervention characteristics, funding sources). Describe any assumptions made about any missing or unclear information. | 9 |
| Study risk of bias assessment | 11 | Specify the methods used to assess risk of bias in the included studies, including details of the tool(s) used, how many reviewers assessed each study and whether they worked independently, and if applicable, details of automation tools used in the process. | 9-10 |
| Effect measures | 12 | Specify for each outcome the effect measure(s) (e.g. risk ratio, mean difference) used in the synthesis or presentation of results. | 10 |
| Synthesis methods | 13a | Describe the processes used to decide which studies were eligible for each synthesis (e.g. tabulating the study intervention characteristics and comparing against the planned groups for each synthesis (item #5)). | 10-11 |
|  | 13b | Describe any methods required to prepare the data for presentation or synthesis, such as handling of missing summary statistics, or data conversions. | 10-11 |
|  | 13c | Describe any methods used to tabulate or visually display results of individual studies and syntheses. | 10-11 |
|  | 13d | Describe any methods used to synthesize results and provide a rationale for the choice(s). If meta-analysis was performed, describe the model(s), method(s) to identify the presence and extent of statistical heterogeneity, and software package(s) used. | 10-11 |
|  | 13e | Describe any methods used to explore possible causes of heterogeneity among study results (e.g. subgroup analysis, meta-regression). | 10-11 |
|  | 13f | Describe any sensitivity analyses conducted to assess robustness of the synthesized results. | 10-11 |
| Reporting bias assessment | 14 | Describe any methods used to assess risk of bias due to missing results in a synthesis (arising from reporting biases). | Not applicable |
| Certainty assessment | 15 | Describe any methods used to assess certainty (or confidence) in the body of evidence for an outcome. | 11 |
| **RESULTS** | | |  |
| Study selection | 16a | Describe the results of the search and selection process, from the number of records identified in the search to the number of studies included in the review, ideally using a flow diagram. | 11 |
|  | 16b | Cite studies that might appear to meet the inclusion criteria, but which were excluded, and explain why they were excluded. | 11 |
| Study characteristics | 17 | Cite each included study and present its characteristics. | 12 |
| Risk of bias in studies | 18 | Present assessments of risk of bias for each included study. | 12-13 |
| Results of individual studies | 19 | For all outcomes, present, for each study: (a) summary statistics for each group (where appropriate) and (b) an effect estimate and its precision (e.g. confidence/credible interval), ideally using structured tables or plots. | 13-15 |
| Results of syntheses | 20a | For each synthesis, briefly summarise the characteristics and risk of bias among contributing studies. | 13-15 |
|  | 20b | Present results of all statistical syntheses conducted. If meta-analysis was done, present for each the summary estimate and its precision (e.g. confidence/credible interval) and measures of statistical heterogeneity. If comparing groups, describe the direction of the effect. | 13-15 |
|  | 20c | Present results of all investigations of possible causes of heterogeneity among study results. | 13-15 |
|  | 20d | Present results of all sensitivity analyses conducted to assess the robustness of the synthesized results. | 13-15 |
| Reporting biases | 21 | Present assessments of risk of bias due to missing results (arising from reporting biases) for each synthesis assessed. | Not applicable |
| Certainty of evidence | 22 | Present assessments of certainty (or confidence) in the body of evidence for each outcome assessed. | 15-16 |
| **DISCUSSION** | | |  |
| Discussion | 23a | Provide a general interpretation of the results in the context of other evidence. | 16-19 |
|  | 23b | Discuss any limitations of the evidence included in the review. | 19 |
|  | 23c | Discuss any limitations of the review processes used. | 20 |
|  | 23d | Discuss implications of the results for practice, policy, and future research. | 20 |
| **OTHER INFORMATION** | | |  |
| Registration and protocol | 24a | Provide registration information for the review, including register name and registration number, or state that the review was not registered. | 7 |
|  | 24b | Indicate where the review protocol can be accessed, or state that a protocol was not prepared. | 7 |
|  | 24c | Describe and explain any amendments to information provided at registration or in the protocol. | 7 |
| Support | 25 | Describe sources of financial or non-financial support for the review, and the role of the funders or sponsors in the review. | 21 |
| Competing interests | 26 | Declare any competing interests of review authors. | 21 |
| Availability of data, code and other materials | 27 | Report which of the following are publicly available and where they can be found: template data collection forms; data extracted from included studies; data used for all analyses; analytic code; any other materials used in the review. | Not applicable |

SUPPLEMENTARY TABLE S2. ELIGIBILITY CRITERIA

| *Criteria* | *Inclusion* | *Exclusion* |
| --- | --- | --- |
| Population | Participants diagnosed with T2DM were 18 years of age or older, regardless of the severity of the condition or mode of treatment (oral or parenteral medication). | - Type 1 diabetes mellitus - Gestational diabetes mellitus |
| Intervention | - Intervention adopted wearable technology. - A wearable device was used as an essential part of the intervention. - The intervention aims to enhance physical activity. | - Invasive wearables - Wearables that dispensed medication - Wearables that were used merely to measure outcome |
| Comparator | - Usual care - Waitlist control - No treatment - Use of wearable devices alone - Coaching or educational advice | - Alternative physical activity interventions as comparator |
| Primary Outcomes | Physical activity outcomes   - Steps per day - Minutes of moderate-vigorous physical activity   Glycemic level outcomes   - HbA1c - Fasting blood glucose |  |
| Secondary Outcomes | - Systolic blood pressure - Diastolic blood pressure - Level of high-density lipoprotein - Level of low-density lipoprotein - Body mass index |  |
| Study Design | Randomized controlled trials | - Other experimental study designs - Non-experimental study designs - Qualitative studies - Review papers - Pilot trials - Feasibility trials - Studies with inaccessible full text |
| Years | No limited |  |
| Language | English |  |
| Publication | Published or not published |  |

SUPPLEMENTARY TABLE S3. FULL SEARCH STRATEGY

| *Concept* | *Search Strategy* | |
| --- | --- | --- |
| *PubMed* | | |
| Diabetes | #1 | Diabetes |
|  | #2 | "Diabetes Mellitus"[Mesh] OR "Diabetes, Gestational"[Mesh] OR "Diabetes Mellitus, Type 2"[Mesh] OR "Diabetes Mellitus, Type 1"[Mesh] |
|  | #3 | #1 OR #2 |
| Wearable-based intervention | #4 | "wearable"[Title/Abstract] OR "wearables"[Title/Abstract] OR "fitness tracker"[Title/Abstract] OR "activity tracker"[Title/Abstract] OR "smartwatch"[Title/Abstract] OR "pedometer"[Title/Abstract] OR "accelerometer"[Title/Abstract] |
|  | #5 | "wearable electronic devices"[MeSH] |
|  | #6 | #4 OR #5 |
|  | #7 | #3 AND #6 |
|  |  | RCT filter |
| Combined search strategy | | (("Diabetes Mellitus"[MeSH Terms] OR "diabetes, gestational"[MeSH Terms] OR "diabetes mellitus, type 2"[MeSH Terms] OR "diabetes mellitus, type 1"[MeSH Terms] OR ("diabete"[All Fields] OR "Diabetes Mellitus"[MeSH Terms] OR ("diabetes"[All Fields] AND "mellitus"[All Fields]) OR "Diabetes Mellitus"[All Fields] OR "diabetes"[All Fields] OR "diabetes insipidus"[MeSH Terms] OR ("diabetes"[All Fields] AND "insipidus"[All Fields]) OR "diabetes insipidus"[All Fields] OR "diabetic"[All Fields] OR "diabetics"[All Fields] OR "diabets"[All Fields])) AND "randomized controlled trial"[Publication Type] AND (("wearable electronic devices"[MeSH Terms] OR "wearable"[Title/Abstract] OR "wearables"[Title/Abstract] OR "fitness tracker"[Title/Abstract] OR "activity tracker"[Title/Abstract] OR "smartwatch"[Title/Abstract] OR "pedometer"[Title/Abstract] OR "accelerometer"[Title/Abstract]) AND "randomized controlled trial"[Publication Type])) AND (randomizedcontrolledtrial[Filter]) |
| *Cochrane* | | |
| Diabetes | #1 | (“diabetes mellitus” OR “diabetes mellitus type 1” OR “diabetes mellitus type 2” OR “gestational diabetes” OR diabetes):ti,ab,kw |
|  | #2 | MeSH descriptor: [Diabetes Mellitus] explode all trees |
|  | #3 | #1 OR #2 |
| Wearable-based intervention | #4 | (“wearable electronic device” OR “wearable” OR “fitness tracker” OR “activity tracker” OR smartwatch OR pedometer OR accelerometer):ti,ab,kw |
|  | #5 | MeSH descriptor: [Fitness Trackers] explode all trees |
|  | #6 | #4 OR #5 |
|  | #7 | #3 AND #6 |
|  |  | RCT filter |
| Combined search strategy | | ((“diabetes mellitus” OR “diabetes mellitus type 1” OR “diabetes mellitus type 2” OR “gestational diabetes” OR diabetes):ti,ab,kw) OR MeSH descriptor: [Diabetes Mellitus] explode all trees) AND ((“wearable electronic device” OR “wearable” OR “fitness tracker” OR “activity tracker” OR smartwatch OR pedometer OR accelerometer):ti,ab,kw) OR MeSH descriptor: [Fitness Trackers] explode all trees) |
| *CINAHL* | | |
| Diabetes | #1 | "diabetes mellitus" OR "diabetes Mellitus type 1" OR "diabetes Mellitus type 2" OR "gestational diabetes" OR diabetes |
|  | #2 | (MH "Diabetes Mellitus, Type 2") OR (MH "Diabetes Mellitus, Type 1+") OR (MH "Diabetes Mellitus, Gestational") OR (MH "Diabetes Mellitus+") |
|  | #3 | #1 OR #2 |
| Wearable-based intervention | #4 | “accelerometer" OR "pedometer" OR "wearable electronic device" OR "activity tracker" OR "smartwatch" |
|  | #5 | (MH "Fitness Trackers") OR (MH "Pedometers") OR (MH "Accelerometers") |
|  | #6 | #4 OR #5 |
|  | #7 | #3 AND #6 |
|  |  | RCT filter |
| Combined search strategy | | ( (MH "Fitness Trackers") OR (MH "Pedometers") OR (MH "Accelerometers") OR "accelerometer" OR "pedometer" OR "wearable electronic device" OR "activity tracker" OR "smartwatch" ) AND ( (MH "Diabetes Mellitus, Type 2") OR (MH "Diabetes Mellitus, Type 1+") OR (MH "Diabetes Mellitus, Gestational") OR (MH "Diabetes Mellitus+") OR "diabetes mellitus" OR "diabetes Mellitus type 1" OR "diabetes Mellitus type 2" OR "gestational diabetes" OR diabetes) ) |
| *Embase* | | |
| Diabetes | #1 | 'diabetes mellitus' OR 'non insulin dependent diabetes mellitus' OR 'insulin dependent diabetes mellitus' OR 'pregnancy diabetes mellitus' |
| Wearable-based intervention | #2 | ‘pedometer’ OR ‘accelerometer’ OR  'wearable device' OR 'wearable computer' OR 'wearable electronic device$' OR 'activity tracker' |
|  | #3 | #1 AND #2 |
|  |  | RCT filter |
| Combined search strategy | | (diabetes:ab,ti OR 'diabetes mellitus':ab,ti OR 'non insulin dependent diabetes mellitus':ab,ti OR 'insulin dependent diabetes mellitus':ab,ti OR 'pregnancy diabetes mellitus':ab,ti) AND (pedometer:ab,ti OR accelerometer:ab,ti OR 'wearable device':ab,ti OR 'wearable computer':ab,ti OR 'wearable electronic device$':ab,ti OR 'activity tracker':ab,ti) AND [randomized controlled trial]/lim |
| *Scopus* | | |
| Diabetes | #1 | diabetes  OR  diabetes  AND mellitus  OR  diabetes  AND mellitus  AND type  2  OR  diabetes  AND mellitus  AND type  1  OR  gestational  AND diabetes |
| Wearable-based intervention | #2 | smartwatch  OR  wearable  AND device  OR  wearable  OR  wearable  AND electronic  AND device  OR  activity  AND tracker  OR  fitness  AND tracker |
|  | #3 | #1 AND #2 |
|  |  | English filter  RCT filter |
| Combined search strategy | | ( TITLE-ABS-KEY ( diabetes  OR  "diabetes mellitus"  OR  "diabetes mellitus type 2"  OR  "diabetes mellitus type 1"  OR  "gestational diabetes" )  AND  TITLE-ABS-KEY ( smartwatch*  OR  "wearable device*"  OR  wearable*  OR  "wearable electronic device*"  OR  "activity tracker*"  OR  "fitness tracker*"  OR  pedometer*  OR  accelerometer* ) )  AND  ( INDEXTERMS ( "clinical trials"  OR  "clinical trials as a topic"  OR  "randomized controlled trial"  OR  "Randomized Controlled Trials as Topic"  OR  "controlled clinical trial"  OR  "Controlled Clinical Trials"  OR  "random allocation"  OR  "Double-Blind Method"  OR  "Single-Blind Method"  OR  "Cross-Over Studies"  OR  "Placebos"  OR  "multicenter study"  OR  "double blind procedure"  OR  "single blind procedure"  OR  "crossover procedure"  OR  "clinical trial"  OR  "controlled study"  OR  "randomization"  OR  "placebo" ) )  OR  ( TITLE-ABS-KEY ( ( "clinical trials"  OR  "clinical trials as a topic"  OR  "randomized controlled trial"  OR  "Randomized Controlled Trials as Topic"  OR  "controlled clinical trial"  OR  "Controlled Clinical Trials as Topic"  OR  "random allocation"  OR  "randomly allocated"  OR  "allocated randomly"  OR  "Double-Blind Method"  OR  "Single-Blind Method"  OR  "Cross-Over Studies"  OR  "Placebos"  OR  "cross-over trial"  OR  "single blind"  OR  "double blind"  OR  "factorial design"  OR  "factorial trial" ) ) )  OR  ( TITLE-ABS ( clinical  AND  trial*  OR  trial*  OR  rct*  OR  random*  OR  blind* ) )  AND  ( LIMIT-TO ( SRCTYPE ,  "j" ) )  AND  ( LIMIT-TO ( DOCTYPE ,  "ar" ) )  AND  ( LIMIT-TO ( SUBJAREA ,  "MEDI" ) )  AND  ( LIMIT-TO ( LANGUAGE ,  "English" ) ) |
| *IEEE* | | |
| Diabetes | #1 | ("Mesh_Terms":diabetes mellitus OR "Mesh_Terms":diabetes mellitus type 1 OR "Mesh_Terms":diabetes mellitus type 2 OR "Mesh_Terms":gestational diabetes OR diabetes) |
| Wearable-based intervention | #2 | ("Mesh_Terms":wearable electronic device* OR "fitness tracker*" OR "activity tracker*" OR smartwatch* OR pedometer* OR accelerometer*) |
|  | #3 | #1 AND #2 |
|  |  | Journal filter |
| Combined search strategy | | (("Mesh_Terms":diabetes mellitus OR "Mesh_Terms":diabetes mellitus type 1 OR "Mesh_Terms":diabetes mellitus type 2 OR "Mesh_Terms":gestational diabetes OR diabetes) AND ("Mesh_Terms":wearable electronic device* OR "fitness tracker*" OR "activity tracker*" OR smartwatch* OR pedometer* OR accelerometer*)) |
| *Web of Science* | | |
| Diabetes | #1 | diabetes OR diabetes mellitus OR diabetes mellitus type 1 OR diabetes mellitus type 2 OR gestational diabetes |
| Wearable-based intervention | #2 | fitness tracker* OR activity tracker* OR wearable electronic device* OR smartwatch* OR pedometer* OR accelerometer* |
|  | #3 | #1 AND #2 |
|  |  | Cochrane’s RCT filter |
| Combined search strategy | | (ALL=(diabetes OR diabetes mellitus OR diabetes mellitus type 1 OR diabetes mellitus type 2 OR gestational diabetes OR non insulin dependent diabetes mellitus OR insulin dependent diabetes mellitus OR pregnancy diabetes mellitus)) AND ALL=(fitness tracker* OR activity tracker* OR wearable electronic device* OR smartwatch* OR pedometer* OR accelerometer*) AND TS=(randomised OR randomized OR randomisation OR randomisation OR placebo* OR (random* AND (allocat* OR assign*)) OR (blind* AND (single OR double OR treble OR triple))) |
| ProQuest Dissertations & Theses Global | | |
| Diabetes | #1 | ABSTRACT,TITLE(diabetes) OR MESH(Diabetes Mellitus OR Diabetes, Gestational OR Diabetes Mellitus, Type 2 OR Diabetes Mellitus, Type 1) |
| Wearable-based intervention | #2 | ABSTRACT,TITLE(wearable* OR fitness tracker* OR activity tracker* OR smartwatch* OR pedometer* OR accelerometer*) OR MESH(wearable electronic devices) |
|  | #3 | #1 AND #2 |
|  |  | English filter |
| Combined search strategy | | (ABSTRACT,TITLE(diabetes) OR MESH(Diabetes Mellitus OR Diabetes, Gestational OR Diabetes Mellitus, Type 2 OR Diabetes Mellitus, Type 1)) AND (ABSTRACT,TITLE(wearable* OR fitness tracker* OR activity tracker* OR smartwatch* OR pedometer* OR accelerometer*) OR MESH(wearable electronic devices)) |

SUPPLEMENTARY TABLE S4. TABLE OF EXCLUDED STUDIES WITH REASONS

|  | *Articles* | *Reasons* |
| --- | --- | --- |
| 1 | Effect of a Behavioral Intervention Strategy for Adoption and Maintenance of a Physically Active Lifestyle: The Italian Diabetes and Exercise Study 2 (IDES_2): A Randomized Controlled Trial (Balducci, 2017) | Wearable to measure outcome only |
| 2 | Walking prescription of 10 000 steps per day in patients with type 2 diabetes mellitus: a randomised trial in Nigerian general practice (Fayehun, 2018) | Wearable to measure outcome only |
| 3 | Increasing Physical Activity in Belgian Type 2 Diabetes Patients: a Three-Arm Randomized Controlled Trial (Greef, 2011) | Wearable to measure outcome only |
| 4 | A daily physical activity and diet intervention for individuals with type 2 diabetes mellitus: a randomized controlled trial (Van Rooijen, 2010) | Wearable to measure outcome only |
| 5 | Effect of a Behavioral Intervention Strategy on Sustained Change in Physical Activity and Sedentary Behavior in Patients With Type 2 Diabetes: the IDES_2 Randomized Clinical Trial (Balducci, 2019) | Wearable to measure outcome only |
| 6 | Relationship between pedometer-registered activity, aerobic capacity and self-reported activity and fitness in patients with type 2 diabetes (Bjørgaas, 2005) | Wearable to measure outcome only |
| 7 | Effectiveness of Digital Medicines to Improve Clinical Outcomes in Patients with Uncontrolled Hypertension and Type 2 Diabetes: prospective, Open-Label, Cluster-Randomized Pilot Clinical Trial (Frias, 2017) | Pilot study |
| 8 | Effect of progressive pedometer based walking intervention on quality of life and general well being among patients with type 2 diabetes (Guglani, 2014) | Wrong outcome |
| 9 | Improved cardiovascular health following a progressive walking and dietary intervention for type 2 diabetes (Johnson, 2009) | Wrong intervention |
| 10 | Non-locomotive physical activity intervention using a tri-axial accelerometer reduces sedentary time in type 2 diabetes (Miyamoto, 2017) | Wrong outcome |
| 11 | Randomized controlled trial for assessment of internet of things system to guide intensive glucose control in diabetes outpatients: nagoya health navigator study (Onoue, 2018) | Conference proceeding; no full text |
| 12 | The effect of automated text messaging and goal setting on pedometer adherence and physical activity in patients with diabetes: a randomized controlled trial (Polgreen, 2018) | Wrong population |
| 13 | Efficacy of a Self-Regulation-Based Electronic and Mobile Health Intervention Targeting an Active Lifestyle in Adults Having Type 2 Diabetes and in Adults Aged 50 Years or Older: two Randomized Controlled Trials (Poppe, 2019) | Wearable to measure outcome only |
| 14 | Effects of lifestyle modifications on patients with type 2 diabetes: the Japan Diabetes Complications Study (JDCS) study design, baseline analysis and three year-interim report (Sone, 2002) | Wearable to measure outcome only |
| 15 | Comparison of the effects of using activity monitors and pedometers in patients with type 2 diabetes completing therapeutic exercise to reduce blood glucose (Toyoda, 2015) | Conference proceeding; no full text |
| 16 | Exercise improves cognitive function – a randomized trial on the effects of physical activity on cognition in type 2 diabetes patients (Leischik, 2021) | Wrong outcome |
| 17 | Effects of a diet with or without physical activity on angiopoietin-like protein 8 concentrations in overweight/obese patients with newly diagnosed type 2 diabetes: a randomized controlled trial (Hu, 2019) | Wearable to measure outcome only |
| 18 | Effects of supported exercise training on health-related quality of life in patients with type 2 diabetes (Timurtas, 2019) | Conference proceeding; no full text |
| 19 | The Alberta Diabetes and Physical Activity Trial (ADAPT): a randomized trial evaluating theory-based interventions to increase physical activity in adults with type 2 diabetes (Plotnikoff, 2013) | Wearable to measure outcome only |
| 20 | Lessened decline in physical activity and impairment of older adults with diabetes with telemedicine and pedometer use: results from the IDEATel study (Weinstock, 2011) | Pedometer not part of main intervention |
| 21 | Daily walking is effective for the management of pregnant women with gestational diabetes mellitus (Hayashi, 2018) | Wearable to measure outcome only |
| 22 | A Daily Physical Activity and Diet Intervention for Individuals with Type 2 Diabetes Mellitus: A Randomized Controlled Trial (Agatha, 2010) | Wearable to measure outcome only |
| 23 | Diet and Exercise among Asian and Pacific Islanders with Type 2 Diabetes (Yomogida, 2013) | No full text |
| 24 | Living Well With Diabetes: 24-Month Outcomes From a Randomized Trial of Telephone-Delivered Weight Loss and Physical Activity Intervention to Improve Glycemic Control (Eakin, 2014) | Wearable to measure outcome only |
| 25 | An intervention to decrease sedentary behavior in older adults: A secondary analysis of a randomized controlled trial (Abraham, 2023) | Wrong population |
| 26 | A Randomized Pilot Trial Using Mobile Health and Financial Incentives to Motivate Heart-Healthy Behaviors in Adolescents With Type 1 Diabetes (Maxwell, 2023) | Wrong outcome |
| 27 | Fit24, a digital health intervention to reduce type 2 diabetes risk among Hispanic youth: Protocol for a feasibility pilot study (Soltero, 2023) | Protocol |
| 28 | Physical activity maintenance among young adult cancer survivors in an mHealth intervention: Twelve-month outcomes from the IMPACT randomized controlled trial (Valle, 2023) | Wrong population |
| 29 | Effectiveness of an intervention to reduce sedentary behaviour as a personalised secondary prevention strategy for patients with coronary artery disease: main outcomes of the SIT LESS randomised clinical trial (van Bakel, 2023) | Wrong population |
| 30 | mHealth intervention delivered in general practice to increase physical activity and reduce sedentary behaviour of patients with prediabetes and type 2 diabetes(ENERGISED): rationale and study protocol for a pragmatic randomised controlled tri (Vetrovsky, 2023) | Protocol |
| 31 | Physical activity, inactivity and sleep during the Diabetes Remission Clinical Trial (DiRECT) (Cassidy et al, 2023) | Wearable to measure outcome only |
| 32 | Pedometers and Text Messaging to Increase Physical Activity (Newton et al, 2009) | Wrong population |
| 33 | One Drop App With an Activity Tracker for Adults With Type 1 Diabetes: Randomized Controlled Trial (Osborn et al, 2020) | Wrong population |
| 34 | Clinical impact of an integrated e‑health system for diabetes self‑management support and shared decision making (POWER2DM): a randomised controlled trial (Ruissen et al, 2023) | Wrong population |

SUPPLEMENTARY TABLE S5. DESCRIPTION OF WEARABLE TECHNOLOGY-BASED PHYSICAL ACTIVITY INTERVENTIONS

| *Author (Year)* | *Intervention* | *Medium & materials* | *Co-interventions* | *Duration* | *Theoretical basis* | *Feedback* | *Follow-up* |
| --- | --- | --- | --- | --- | --- | --- | --- |
| Alghafri et al. (2018) | ‘MOVEdiabetes’  Self-motivation & monitoring | In-person consultations + WhatsApp messages | 3 personalized individual PA consultations + goal setting | 1 year | Health Belief Model, Stages of Change Model, Social cognitive theory | Daily step count + during consultations + WhatsApp messages | 3 & 12 months |
| Andrews et al. (2011) | ‘Early Activity in Diabetes’ | In-person nurse appointments + PA logs | 3 monthly diet consultation + monthly nurse support + PA program (pedometer-based) + goal setting | 1 year | NR | Daily step count + during appointments | 6 & 12 months |
| Araiza et al. (2006) | Pedometer-based PA program | PA logs | 10,000 steps/day prescription | 6 weeks | NR | Daily step count | 6 weeks |
| Bjørgaas et al. (2008 | PA intervention study | PA logs + nurse visits | Goal setting + nurse visits (strategies to increase walking) | 6 months | NR | Daily step count + during nurse visits | 1, 3 & 6 months |
| Diedrich et al. (2010) | Self-help PA program | In-person appointments with Certified Diabetes Educators + PA logs | Diabetes self-management education program (PA, healthy eating, medication management, glucose monitoring, problem-solving, reducing the risk of complications, psychosocial adjustment) + PA program (pedometer-based) + book handout about pedometers | 3 months | NR | Daily step count | 3 months |
| Engel & Lindner (2006) | Coaching intervention | PA logs + 6 in-person visits + phone calls | Coaching (education, goal setting, motivational strategies to increase walking) | 6 months | NR | Daily step count + during in-person visits + phone calls | 3 & 6 months |
| Greef et al. (2011) | Pedometer-based behavioural modification program with telephone support | In-person session + phone calls + PA log | 1x In-person session (motivational interview, individualised lifestyle plan) + 7x CBT-based telephone support sessions + goal setting | 24 weeks | NR | Daily step count + phone calls | 24 weeks & 1 year |
| Kempny et al. (2008) | - | Device internal memory | Oral advice to increase PA from Dr + goal setting | 5 weeks | NR | Daily step count | 5 weeks |
| Kooiman et al. (2018) | Online self-tracking (eHealth) program | Nurse visits + online +  Device internal memory | DM nurse visits (advice and monitoring) + online lifestyle program (goal setting, weekly PA & diet information, tailored feedback messages) | 13 weeks | Social cognitive theory | Daily step count + DM nurse visits + weekly tailored feedback messages | 13 weeks |
| Li et al. (2021) | mHealth App and Chest-Wearable Remote Exercise Monitoring Intervention | R Plus Health app | Prescribed individualised exercise + goal setting + mHealth fitness app (exercise videos sent via app) + telephone follow ups | 3 months | NR | Feedback from device + monthly telephone follow ups | 1, 2 & 3 months |
| Lystrup et al. (2020) | Fitbit + friends | Mobile application + WhatsApp | Virtual fitness group (compared step count by a leaderboard) + WhatsApp messaging (communicate within group & with research coordinator) + goal setting | 6 months | NR | Daily step count + feedback from research coordinator | 3 & 6 months |
| Matsushita et al. (2022) | - | In-person PT sessions + device internal memory | Exercise instructions from PT + goal setting | 8 weeks | The transtheoretical model | Daily PA levels | 8 weeks |
| Miyauchi et al. (2016) | Exercise Therapy | PA log | Goal setting + exercise instructions pamphlet | 6 months | NR | Daily PA levels | 2 & 6 months |
| Oliveira et al. (2022) | DASH diet vs. DASH diet plus physical activity | Monthly visits + device internal memory | (DASHPED) dietary guidance + encouragement to walk with a pedometer + monthly visits (monitoring, guideline reinforcement, assessed adherence, provided ingredients) + goal setting | 16 weeks | NR | Daily step count + monthly visits | 16 weeks |
| Patel et al. (2021) | Behaviourally Designed Gamification with Social Incentives | Way to Health platform (for remote data collection) + Withings Healthmate application | Gamification to enhance (1) support / (2) collaboration / (3) competition + goal setting | 1 year | NR | Daily step count + email / text message + application | 6 & 12 months |
| Piette et al. (2011) | Telephone Counselling plus Walking | Phone call + manual (about depression CBT, diabetes self-care & PA) + device internal memory | Weekly nurse-delivered telephone CBT program + walking program (pedometer-based) + goal setting | 12 months | NR | Phone calls | 12 months |
| Shenoy et al. (2010) | Aerobic walking program | Heart rate monitor (exercise intensity) + PA log | Walking program (given a walking schedule) + goal setting | 8 weeks | NR | Heart rate monitor + daily step count | 8 weeks |
| Timurtas et al. (2022) | Technology-based and supervised exercise interventions | Mobile app (DIABETEX platform) | Prescribed exercise training (by PT) + mobile app | 12 weeks | The transtheoretical model | Application + smartwatch | 12 weeks |
| Tudor-locke et al. (2004) | First Step Program | Group meetings + PA logs | Goal setting + 4 weekly group meetings + first step program (behaviour modification program, self-monitoring) | 16 weeks | Self-efficacy, social support | Group meetings | 16 & 24 weeks |

CBT, Cognitive-behavioral therapy; DASH, Dietary Approaches to Stop Hypertension; DM, Diabetes mellitus; NR, Not reported; PA, Physical activity; POWER2DM, PT, physical therapists

SUPPLEMENTARY TABLE S6. GRADE SUMMARY TABLE

| **Certainty assessment** | | | | | | | **№ of patients** | | **Effect** | **Certainty** | **Importance** |
| --- | --- | --- | --- | --- | --- | --- | --- | --- | --- | --- | --- |
| **№ of studies** | **Study design** | **Risk of bias** | **Inconsistency** | **Indirectness** | **Imprecision** | **Other considerations** | **Wearable technology-based physical activity interventions** | **Comparators** | **Hedges’ (95% CI)** |  |  |
| **Glycemic levels: HbA1c** | | | | | | | | | | | |
| 20 arms in 17 | randomized trials | serious^a^ | serious^b^ | serious^c^ | serious^d^ | No publication^e^ | 1203 | 844 | g=-0.05  (-0.22, 0.12) | ⨁◯◯◯ Very low | IMPORTANT |
| **Glycemic levels: Fasting blood glucose** | | | | | | | | | | | |
| 6 | randomized trials | serious^a^ | serious^b^ | serious^c^ | serious^d^ | Not applicable | 111 | 113 | g=-0.34  (-1.50, 0.81) | ⨁◯◯◯ Very low | IMPORTANT |
| **Physical Activity: Step per day** | | | | | | | | | | | |
| 11 arms in 9 | randomized trials | serious^a^ | serious^b^ | serious^c^ | serious^d^ | No publication^e^ | 647 | 429 | *g* =0.55  (0.18, 0.90) | ⨁◯◯◯ Very low | IMPORTANT |
| **Physical Activity: Moderate-vigorous physical activity** | | | | | | | | | | | |
| 2 | randomized trials | serious^a^ | no serious | serious^c^ | serious^d^ | Not applicable | 303 | 129 | g=0.34  (-1.41, 2.09) | ⨁◯◯◯ Very low | IMPORTANT |
| **Others: Systolic blood pressure** | | | | | | | | | | | |
| 11 | randomized trials | serious^a^ | no serious | serious^c^ | serious^d^ | No publication^e^ | 649 | 509 | g=-0.15  (-0.30, 0.00) | ⨁◯◯◯ Very low | IMPORTANT |
| **Others: Diastolic blood pressure** | | | | | | | | | | | |
| 11 | randomized trials | serious^a^ | serious^b^ | serious^c^ | serious^d^ | No publication^e^ | 649 | 509 | g=-0.13  (-0.52, 0.26) | ⨁◯◯◯ Very low | IMPORTANT |
| **Others: Body mass index** | | | | | | | | | | | |
| 9 | randomized trials | serious^a^ | no serious | serious^c^ | serious^d^ | Not applicable | 529 | 371 | g=-0.18  (-0.32, -0.03) | ⨁◯◯◯ Very low | IMPORTANT |
| **Others: High-density lipoprotein** | | | | | | | | | | | |
| 7 | randomized trials | serious^a^ | no serious | serious^c^ | serious^d^ | Not applicable | 556 | 410 | g=0.05  (-0.08, 0.19) | ⨁◯◯◯ Very low | IMPORTANT |
| **Others: Low-density lipoprotein** | | | | | | | | | | | |
| 7 | randomized trials | serious^a^ | no serious | serious^c^ | serious^d^ | Not applicable | 666 | 327 | g=-0.06  (-0.16, 0.05) | ⨁◯◯◯ Very low | IMPORTANT |

**CI:** confidence interval

#### Explanations

a. >50% of articles overall risk unclear/high risk

b. Heterogeneity > 50%

c. Different types of wearables and comparators were used

d. Small sample size or/and wide confidence intervals

e. Egger's test *p* < 0.05

**Intention-to-treat**

| Study ID | D1 | D2 | D3 | D4 | D5 | Overall |
| --- | --- | --- | --- | --- | --- | --- |
| Alghafri et al. (2018) |  |  |  |  |  |  |
| Andrews et al. (2011) |  |  |  |  |  |  |
| Greef et al. (2011) |  |  |  |  |  |  |
| Kooiman et al. (2018) |  |  |  |  |  |  |
| Patel et al. (2021) |  |  |  |  |  |  |
| Piette et al. (2011) |  |  |  |  |  |  |
| Timurtas et al. (2022) |  |  |  |  |  |  |

**Per-protocol**

| Study ID | | D1 | D2 | D3 | D4 | D5 | Overall |
| --- | --- | --- | --- | --- | --- | --- | --- |
| Araiza et al. (2006) | |  |  |  |  |  |  |
| Bjørgaas et al. (2008) | |  |  |  |  |  |  |
| Diedrich et al. (2010) | |  |  |  |  |  |  |
| Engel & Lindner (2006) | |  |  |  |  |  |  |
| Kempny et al. (2008) | |  |  |  |  |  |  |
| Li et al. (2021) | |  |  |  |  |  |  |
| Lystrup et al. (2020) | |  |  |  |  |  |  |
| Matsushita et al. (2022) | |  |  |  |  |  |  |
| Miyauchi et al. (2016) | |  |  |  |  |  |  |
| Oliveira et al. (2022) | |  |  |  |  |  |  |
| Shenoy et al. (2010) | |  |  |  |  |  |  |
| Tudor-locke et al. (2004) | |  |  |  |  |  |  |
| D1 | Randomisation process | | | | \|  \| \| --- \| | Low risk | |
| D2 | Deviations from the intended interventions | | | | \|  \| \| --- \| | Some concerns | |
| D3 | Missing outcome data | | | | \|  \| \| --- \| | High risk | |
| D4 | Measurement of the outcome | | | |  |  | |
| D5 | Selection of the reported result | | | |  |  | |

**SUPPLEMENTARY FIG. S1.** RISK OF BIAS SUMMARY.


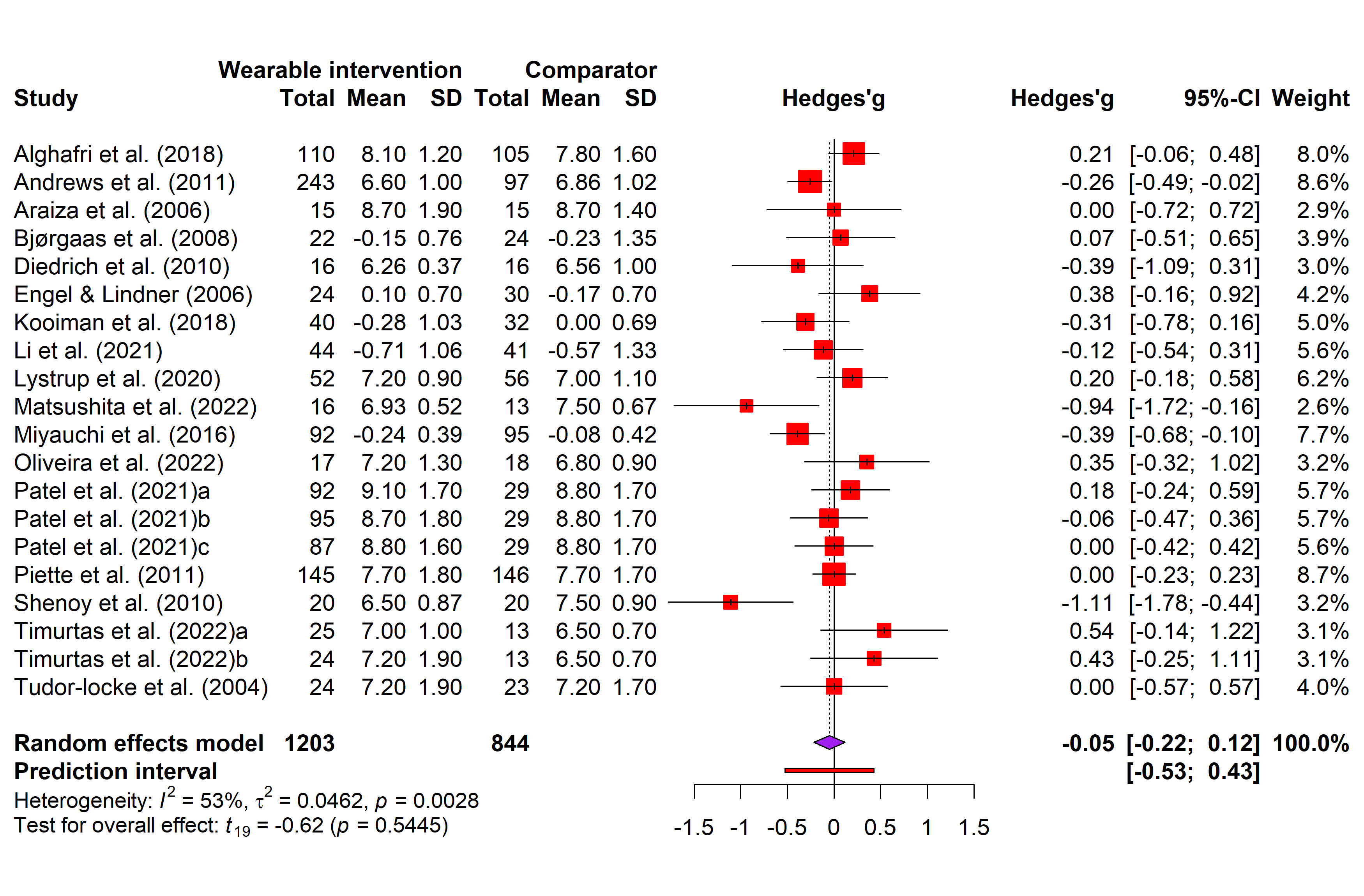


**SUPPLEMENTARY FIG. S2** FOREST PLOT OF EFFECT SIZE ON HBA1C FOR WEARABLE TECHNOLOGY-BASED PHYSICAL ACTIVITY INTERVENTIONS AND COMPARATORS.

**Mean Difference**


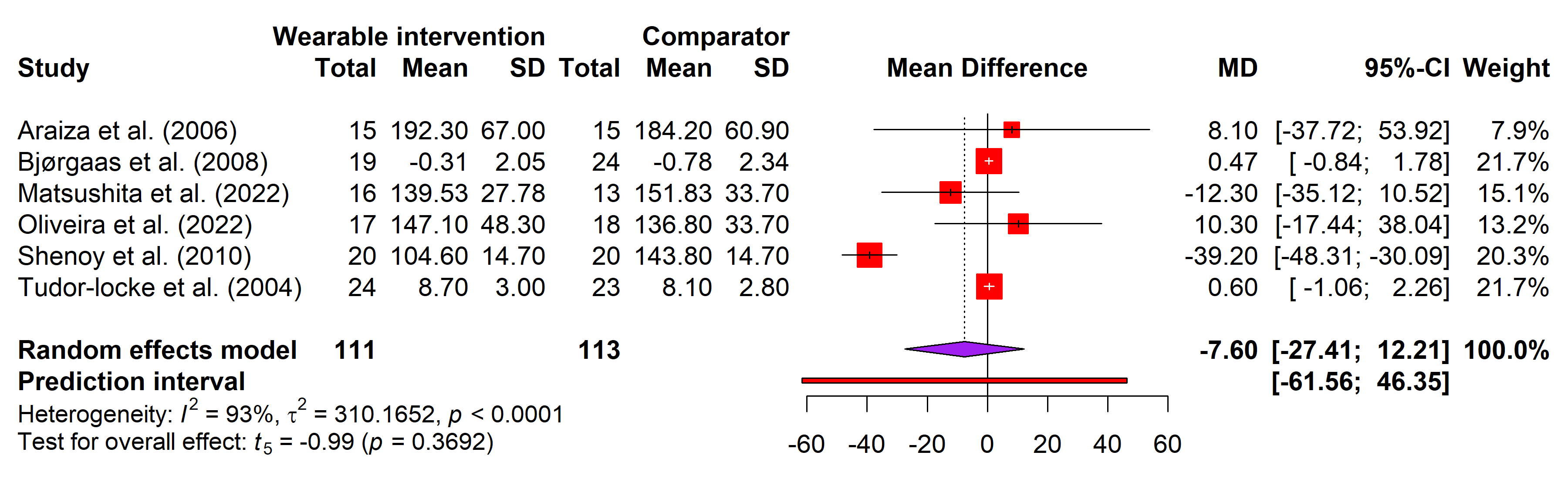


**Effect size (Hedges’*g*)**


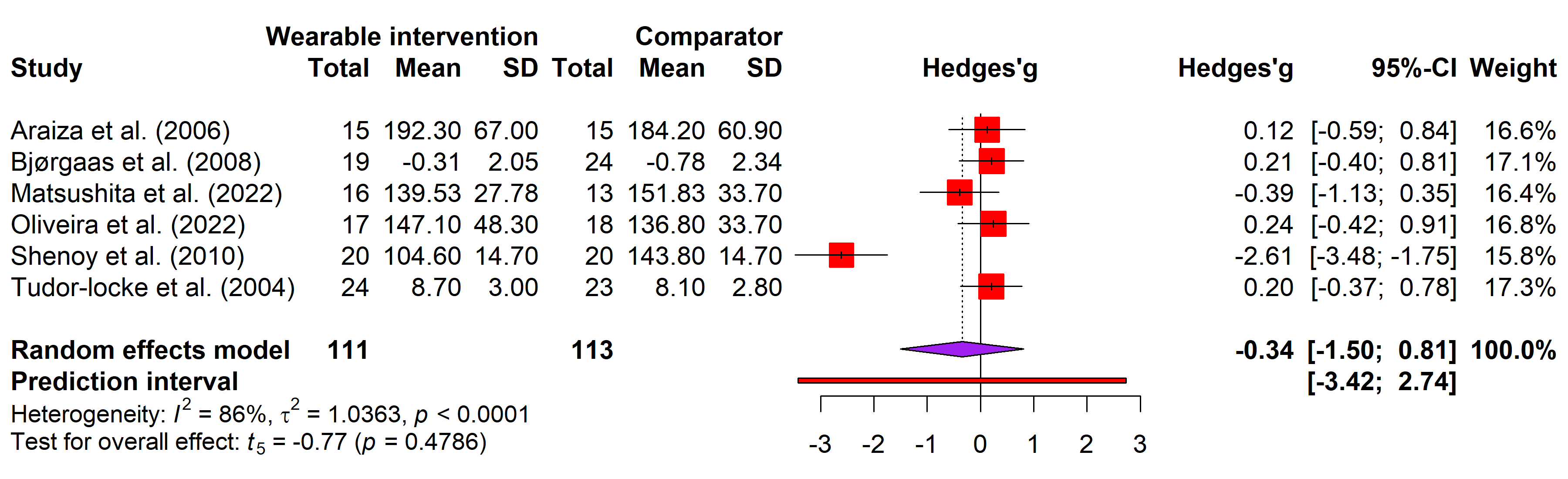


**SUPPLEMENTARY FIG. S3.** FOREST PLOT OF MEAN DIFFERENCE AND EFFECT SIZE ON FASTING BLOOD GLUCOSE FOR WEARABLE TECHNOLOGY-BASED PHYSICAL ACTIVITY INTERVENTIONS AND COMPARATORS.


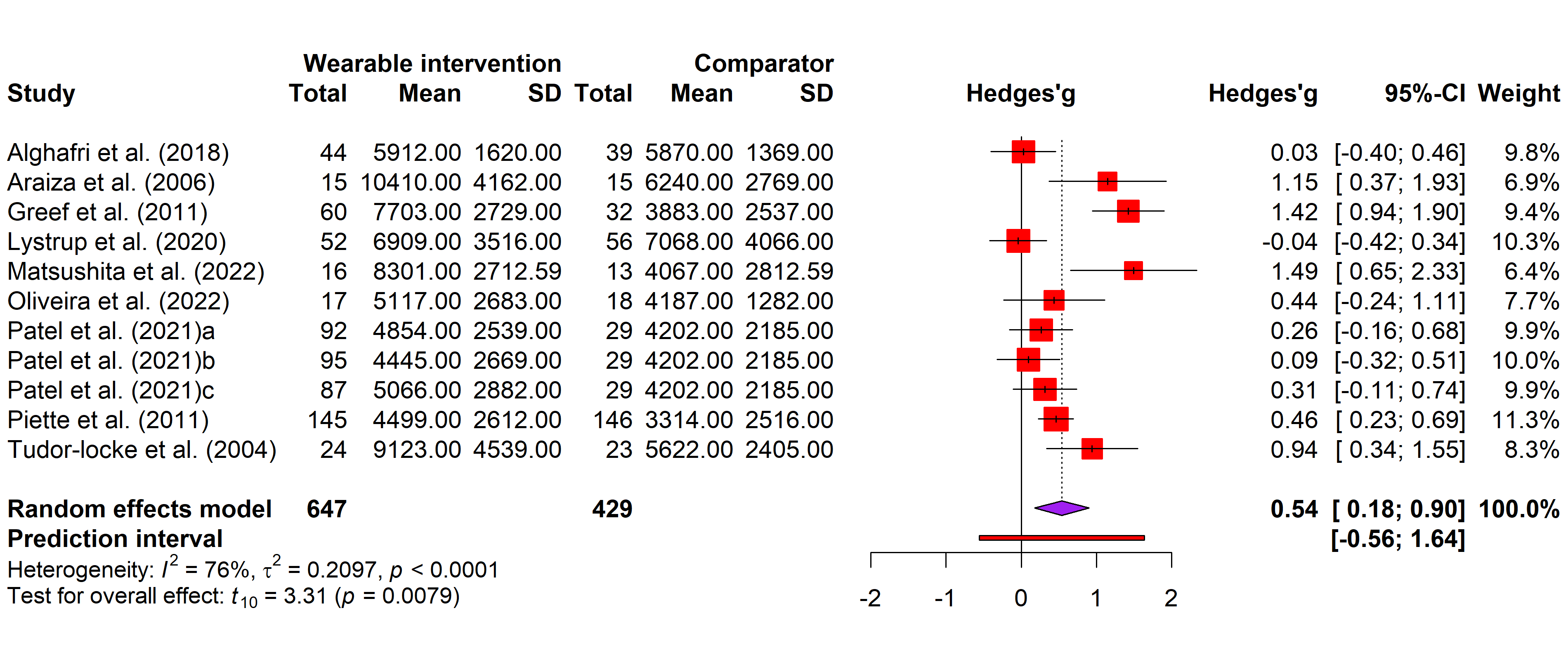


**SUPPLEMENTARY FIG. S4** FOREST PLOT OF EFFECT SIZE ON STEP PER DAY FOR WEARABLE TECHNOLOGY-BASED PHYSICAL ACTIVITY INTERVENTIONS AND COMPARATORS.

**Mean Difference**


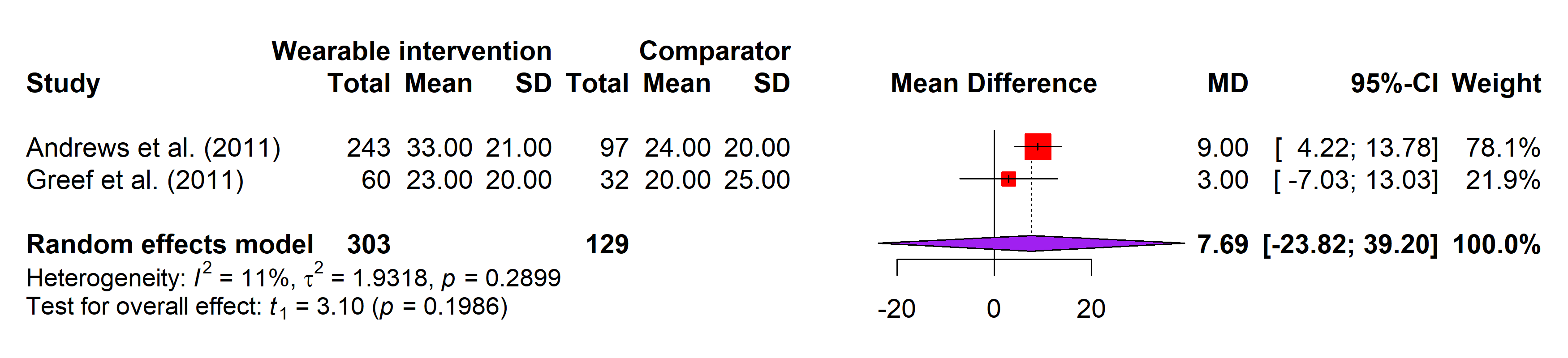


**Effect size (Hedges’*g*)**


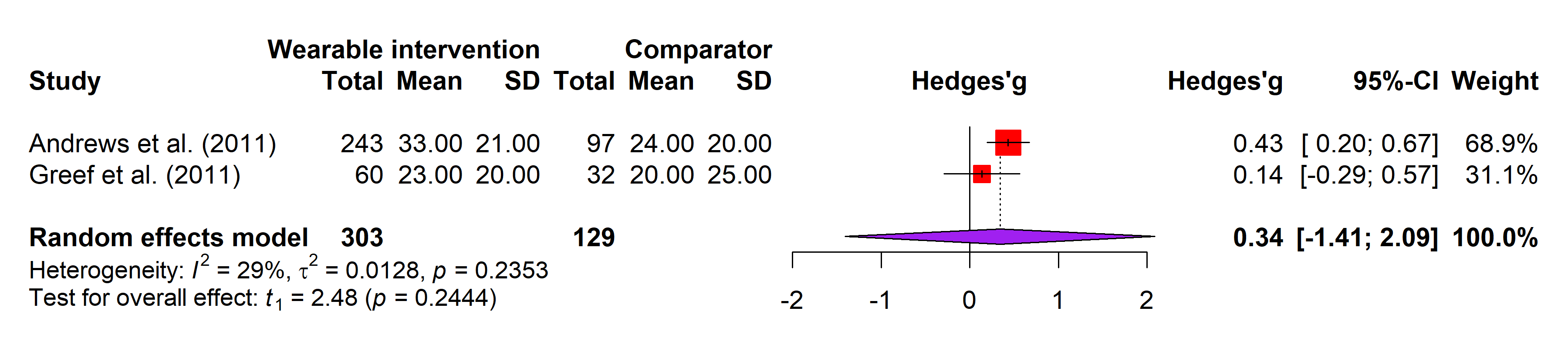


**SUPPLEMENTARY FIG. S5** FOREST PLOT OF MEAN DIFFERENCE AND EFFECT SIZE ON MODERATE-VIGOROUS PHYSICAL ACTIVITY FOR WEARABLE TECHNOLOGY-BASED PHYSICAL ACTIVITY INTERVENTIONS AND COMPARATORS.

**Mean Difference**

**
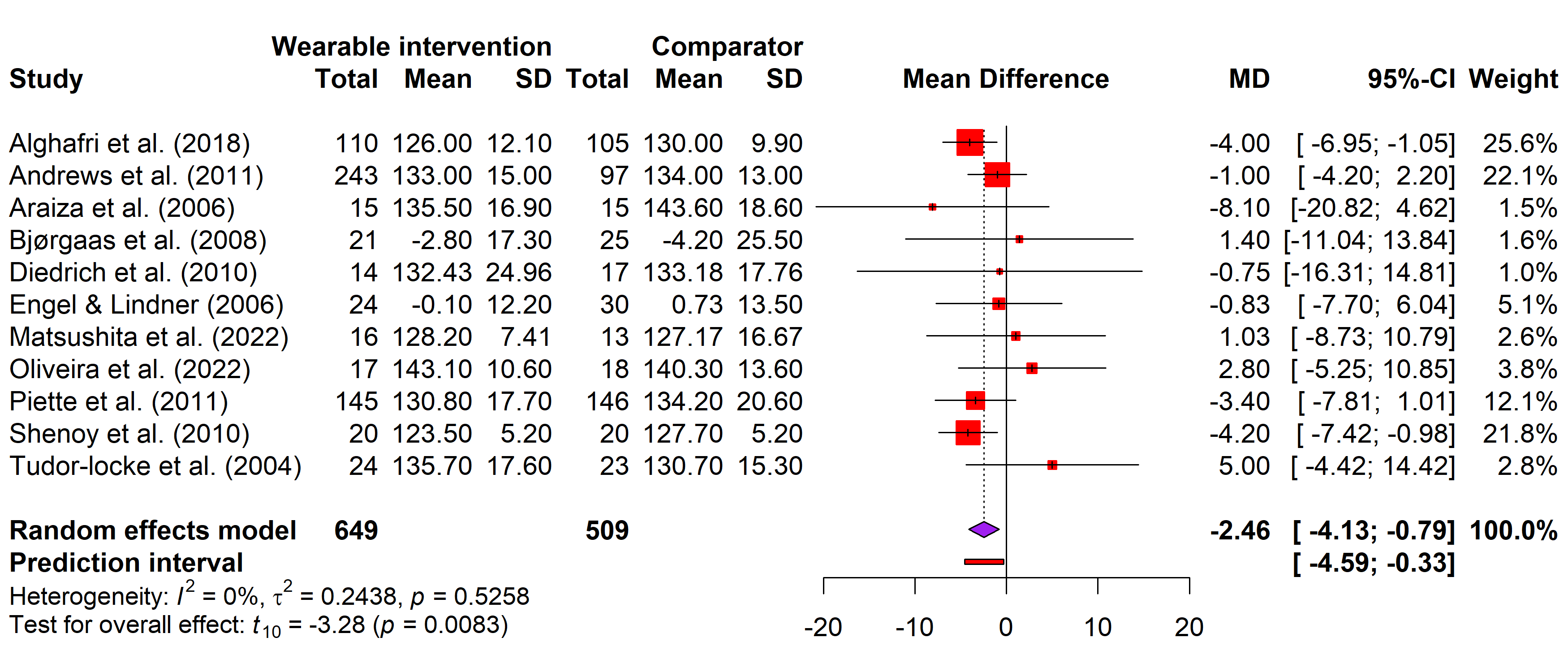
**

**Effect size (Hedges’*g*)**

**
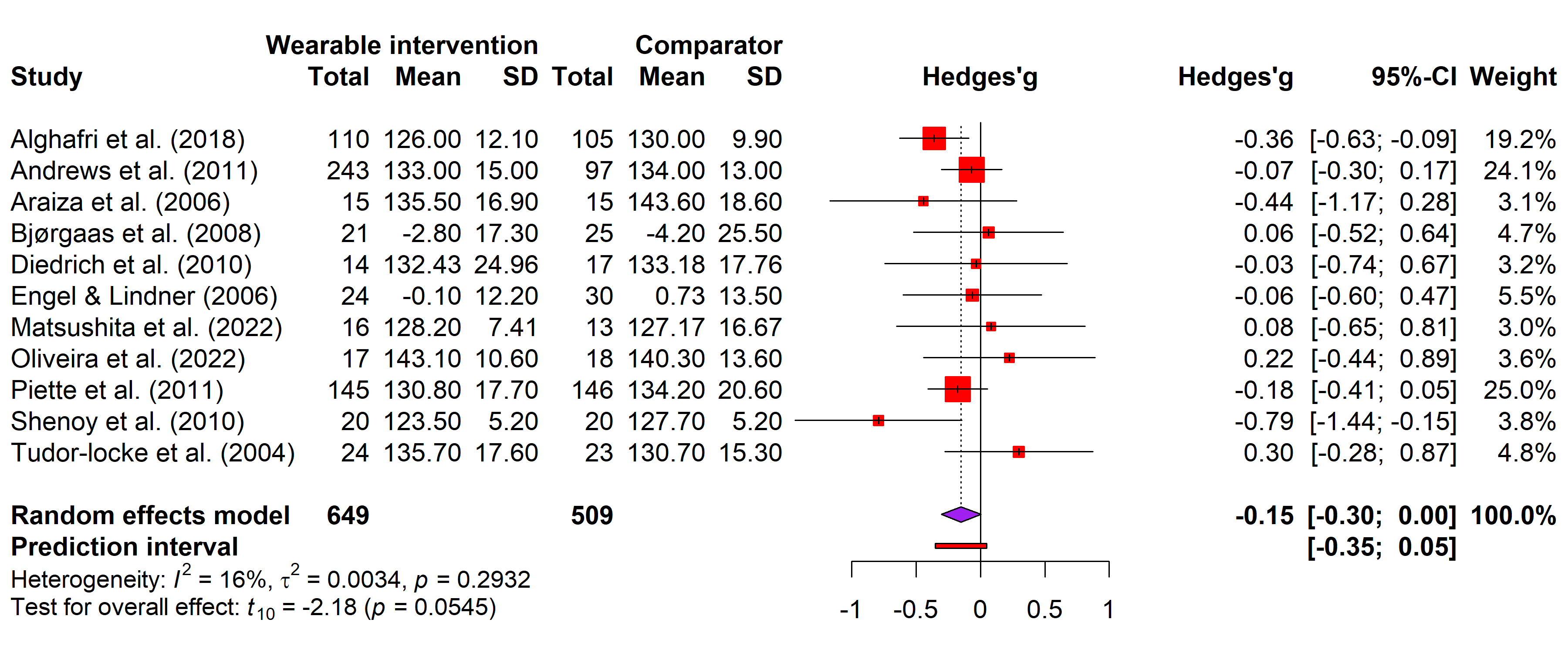
**

**SUPPLEMENTARY FIG. S6**. FOREST PLOT OF MEAN DIFFERENCE AND EFFECT SIZE ON SYSTOLIC BLOOD PRESSURE FOR WEARABLE TECHNOLOGY-BASED PHYSICAL ACTIVITY INTERVENTIONS AND COMPARATORS.

**Mean Difference**

**
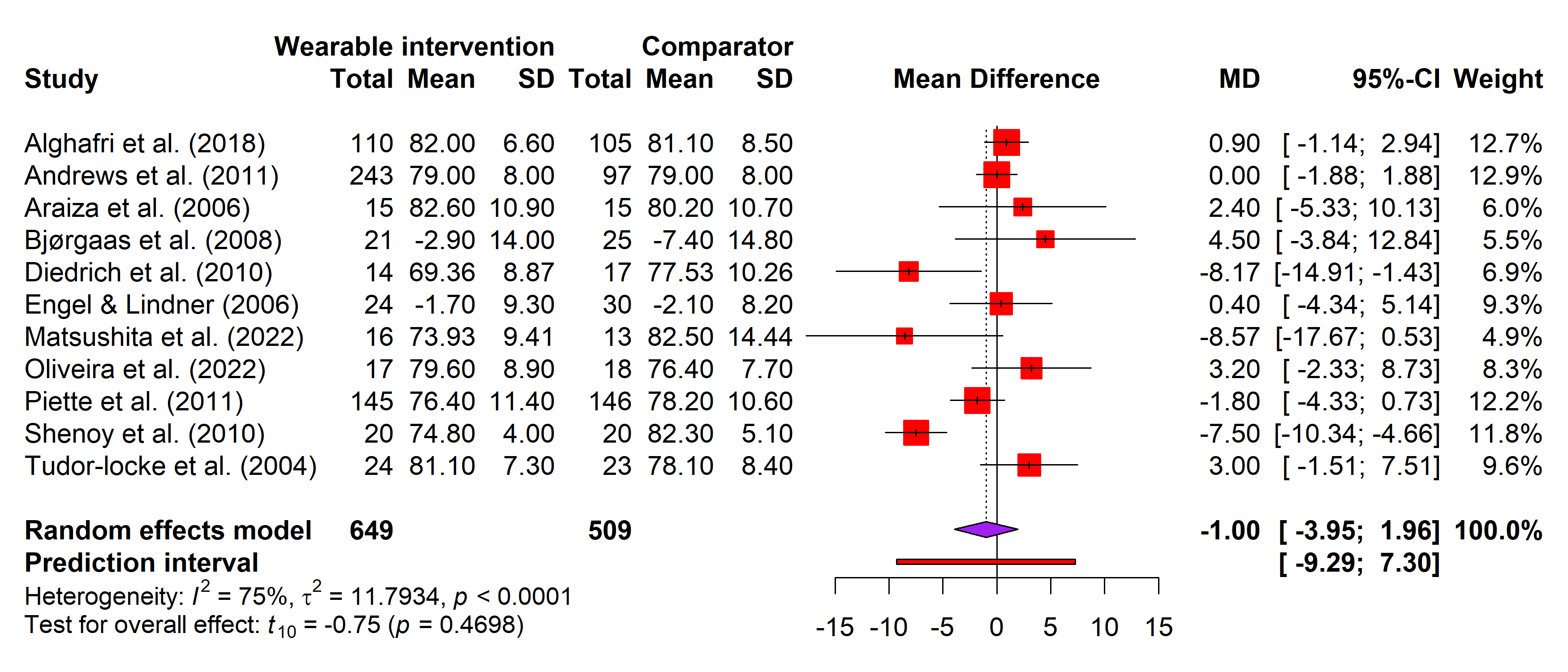
**

**Effect size (Hedges’*g*)**

**
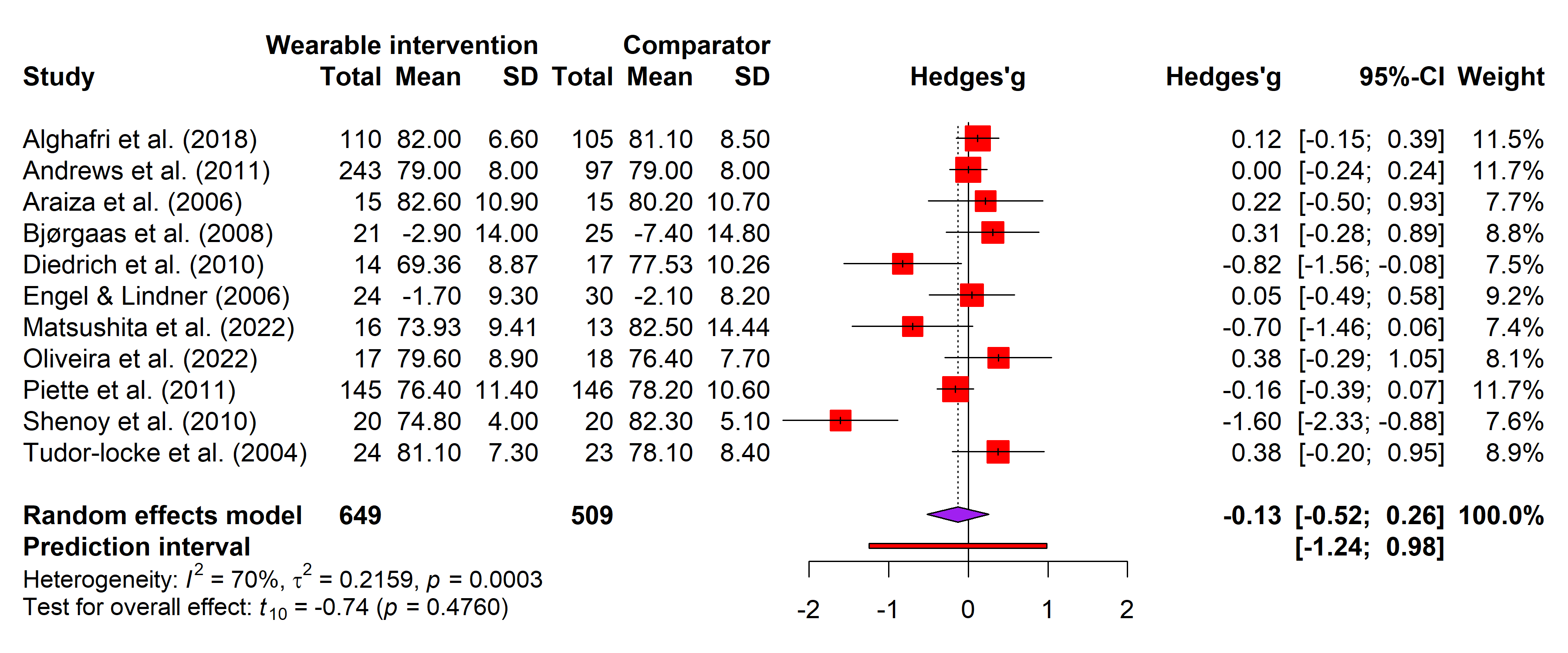
**

**SUPPLEMENTARY FIG. S7**. FOREST PLOT OF MEAN DIFFERENCE AND EFFECT SIZE ON DIASTOLIC BLOOD PRESSURE FOR WEARABLE TECHNOLOGY-BASED PHYSICAL ACTIVITY INTERVENTIONS AND COMPARATORS.

**Mean Difference**

**
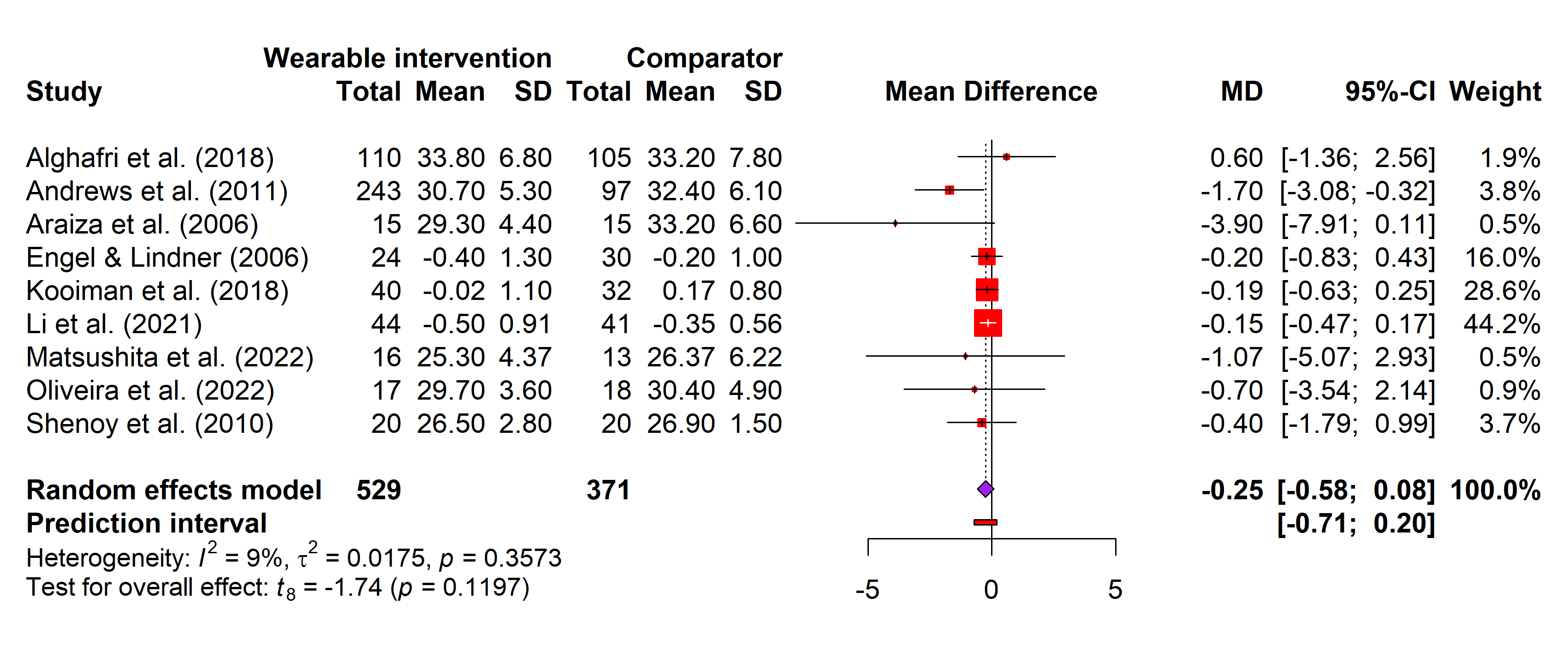
**

**Effect size (Hedges’*g*)**

**
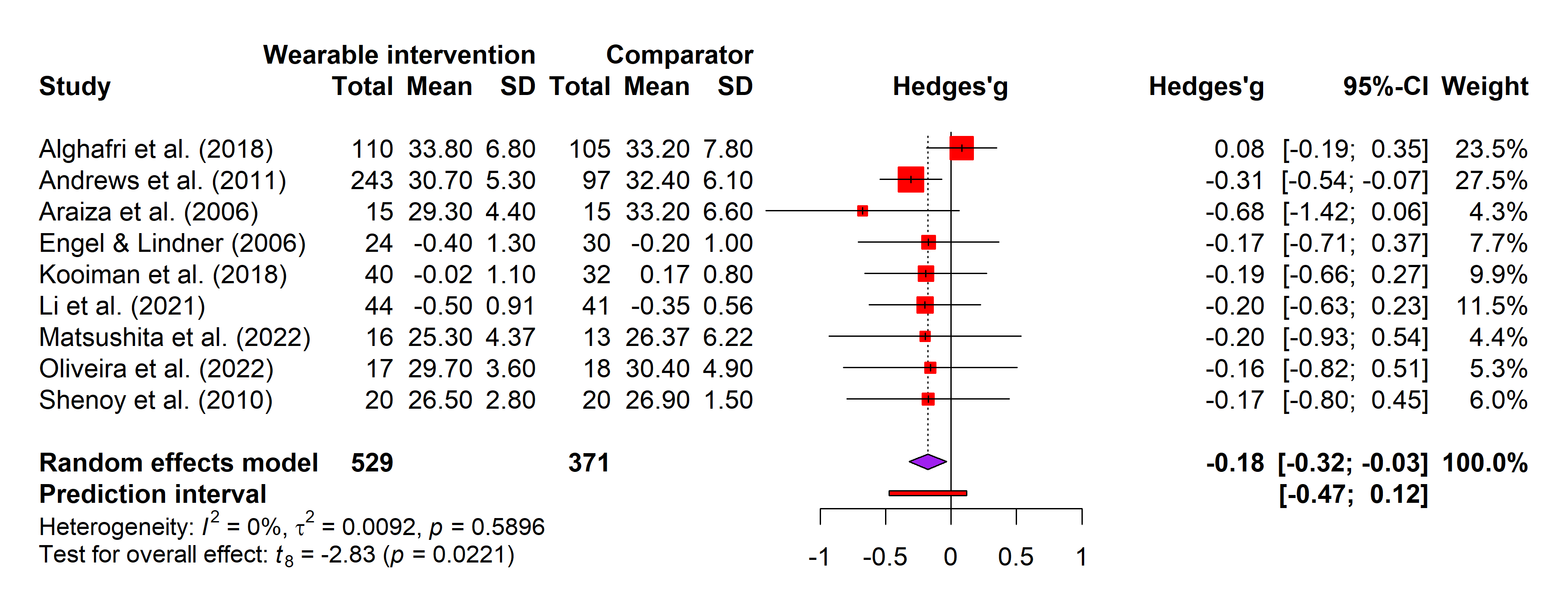
**

**SUPPLEMENTARY FIG. S8.** FOREST PLOT OF MEAN DIFFERENCE AND EFFECT SIZES ON BODY MASS INDEX FOR WEARABLE TECHNOLOGY-BASED PHYSICAL ACTIVITY INTERVENTIONS AND COMPARATORS.

**Mean Difference**

**
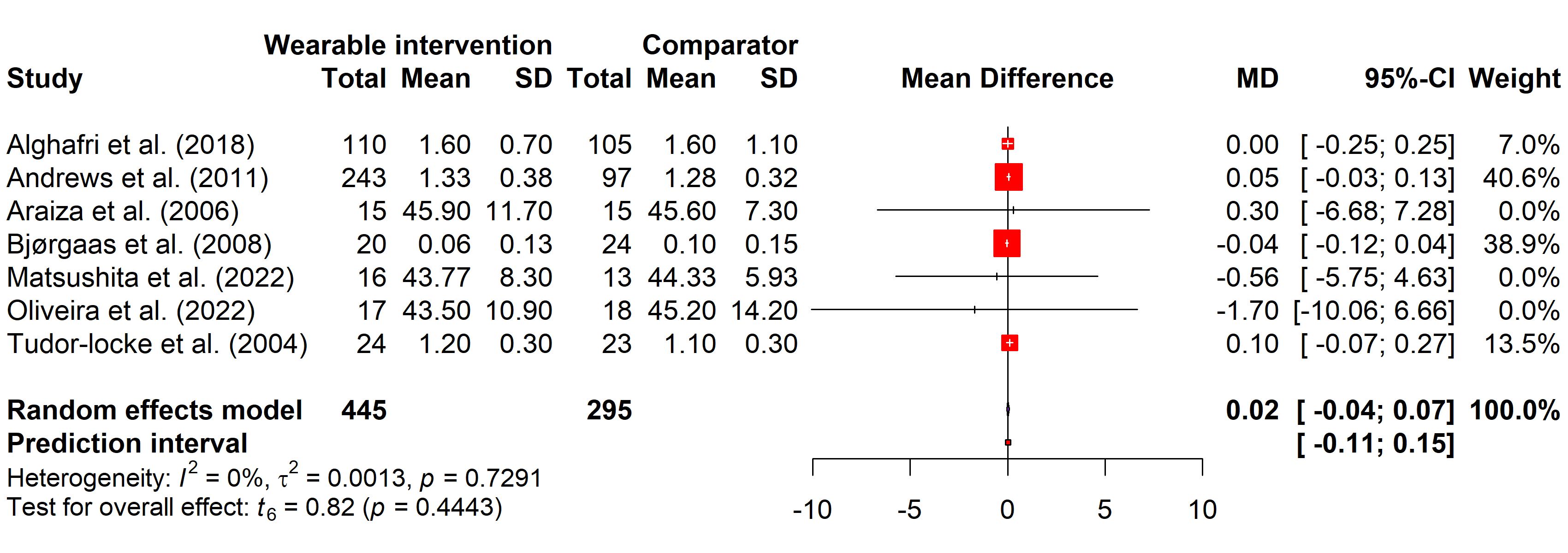
**

**Effect size (Hedges’*g*)**


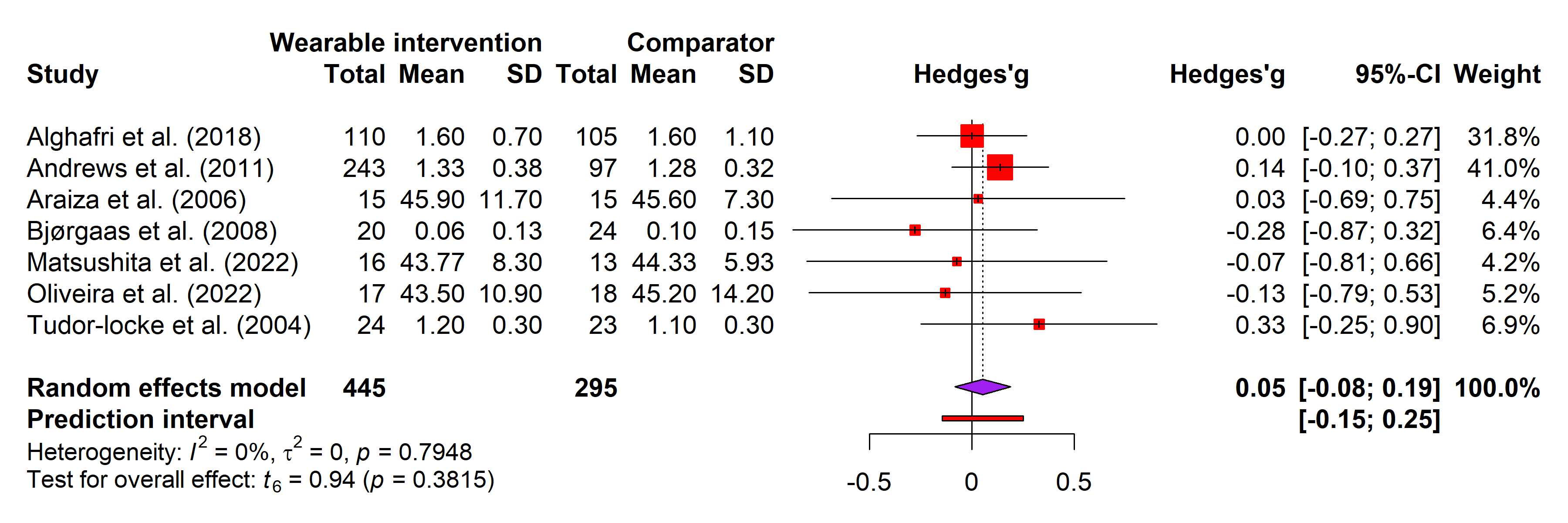
**SUPPLEMENTARY FIG. S9.** FOREST PLOT OF MEAN DIFFRENCE AND EFFECT SIZE ON HIGH-DENSITY LIPOPROTEIN FOR WEARABLE TECHNOLOGY-BASED PHYSICAL ACTIVITY INTERVENTIONS AND COMPARATORS.

**Mean Difference**

**
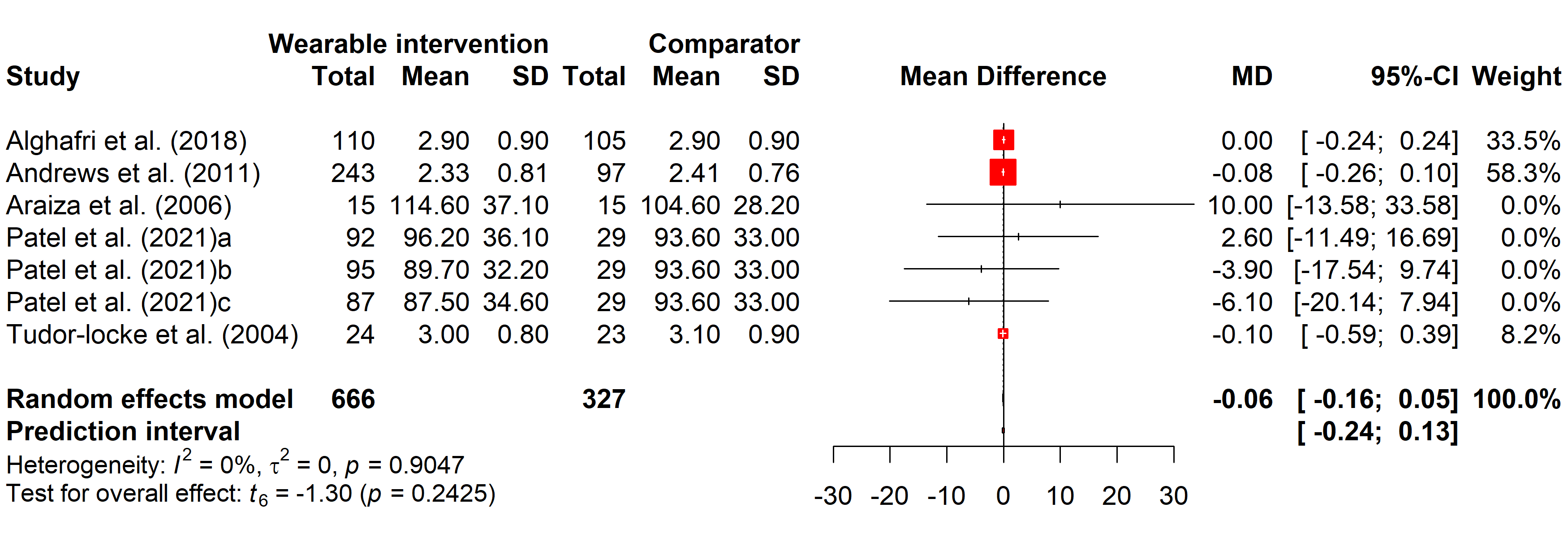
**

**Effect size (Hedges’*g*)**


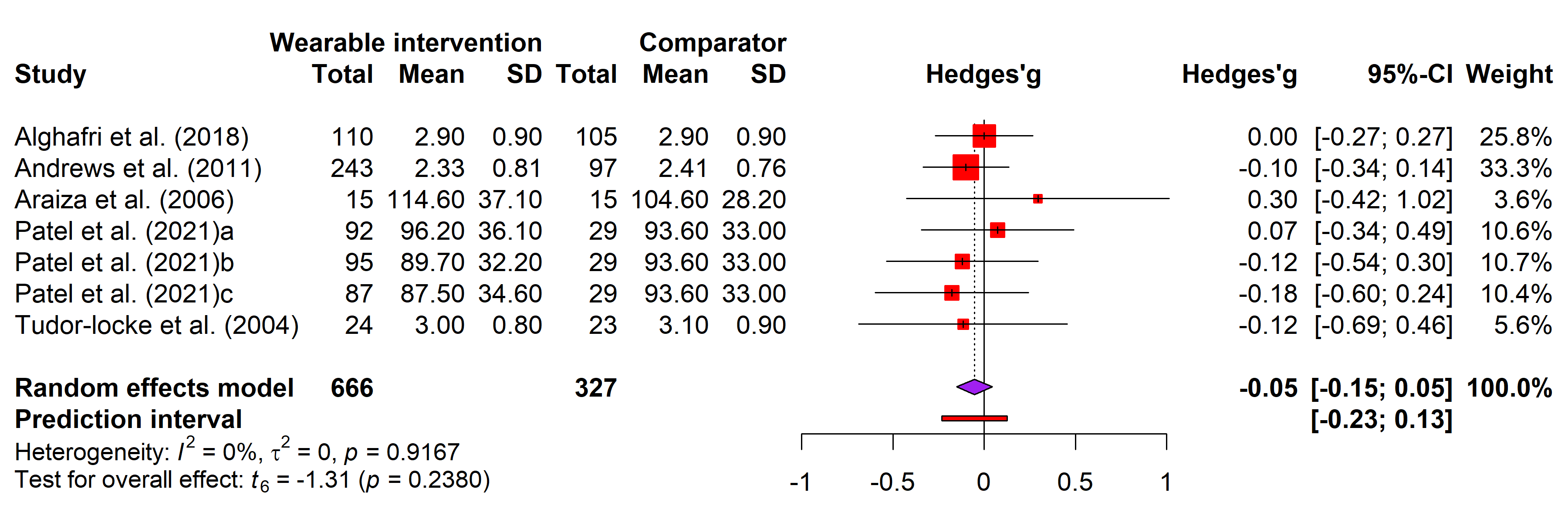


**SUPPLEMENTARY FIG. S10.** FOREST PLOT OF MEAN DIFFERENCE AND EFFECT SIZE ON LOW-DENSITY LIPOPROTEIN FOR WEARABLE TECHNOLOGY-BASED PHYSICAL ACTIVITY INTERVENTIONS AND COMPARATORS.


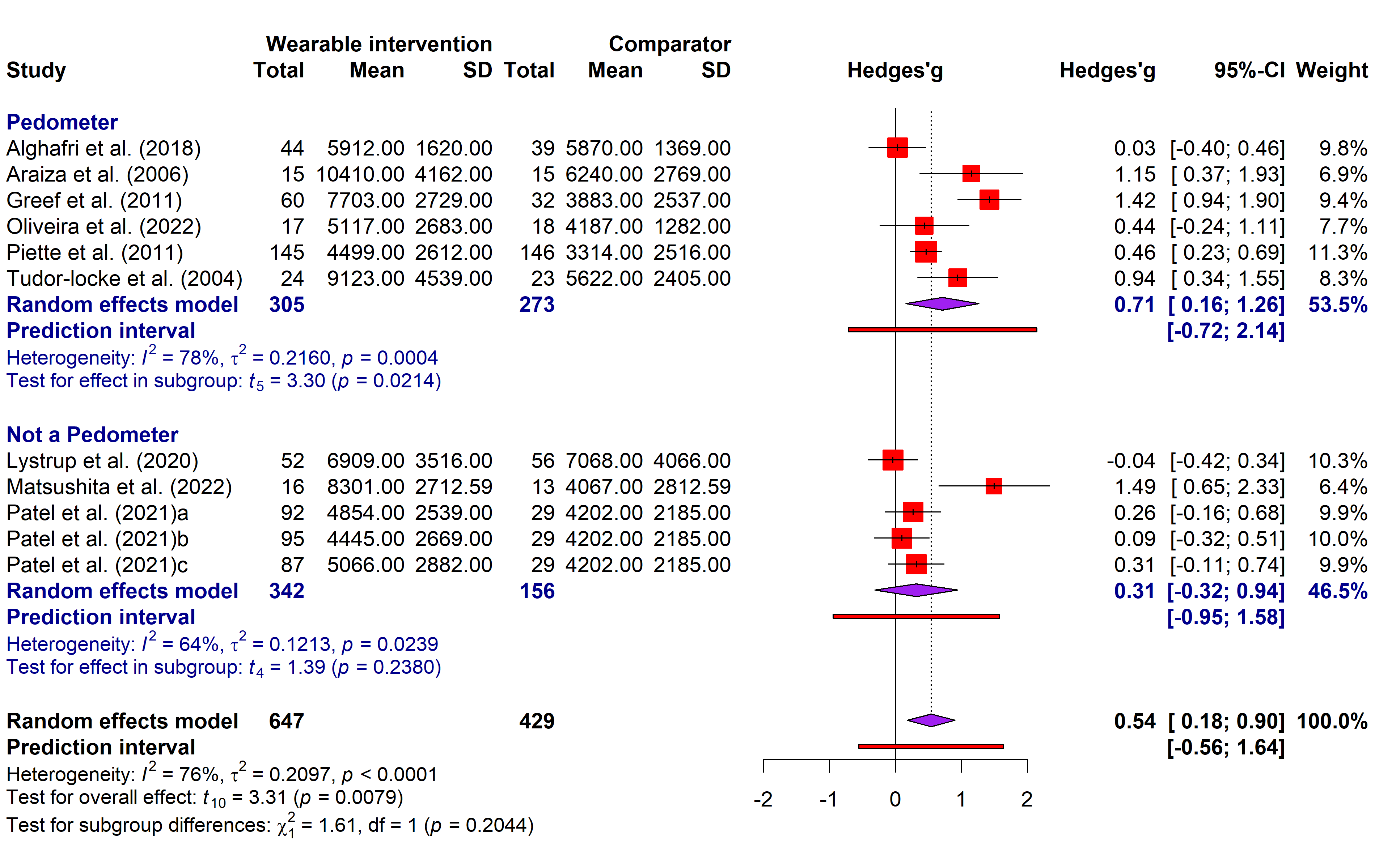


**SUPPLEMENTARY FIG. S11.** SUBGROUP ANALYSIS OF WEARABLE TECHNOLOGY-BASED PHYSICAL ACTIVITY INTERVENTIONS ON STEPS PER DAY BY TYPE OF WEARABLE.


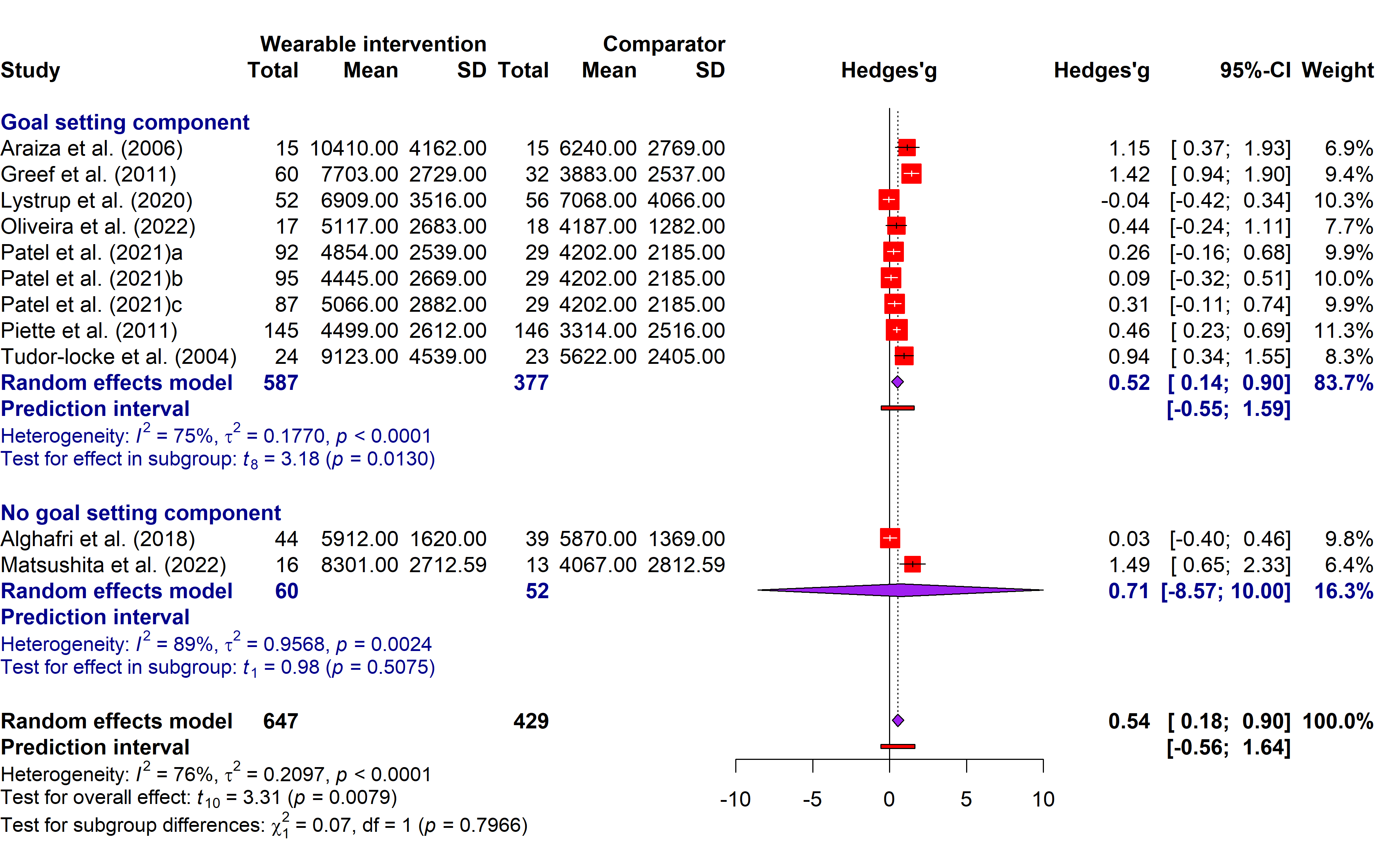


**SUPPLEMENTARY FIG. S12.** SUBGROUP ANALYSIS OF WEARABLE TECHNOLOGY-BASED PHYSICAL ACTIVITY INTERVENTIONS ON STEPS PER DAY BY GOAL SETTING.


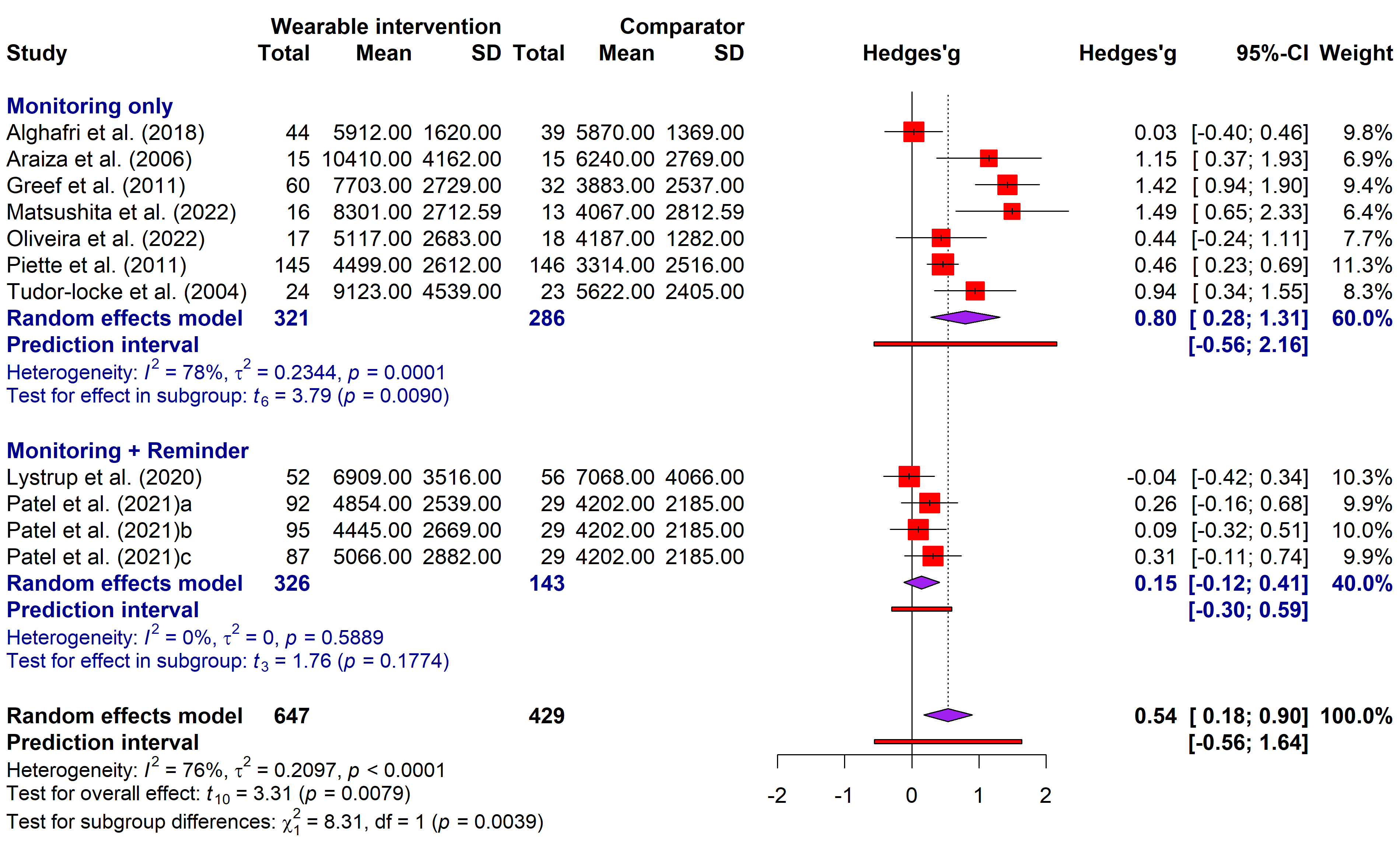


**SUPPLEMENTARY FIG. S13.** SUBGROUP ANALYSIS OF WEARABLE TECHNOLOGY-BASED PHYSICAL ACTIVITY INTERVENTIONS ON STEPS PER DAY BY FUNCTION.


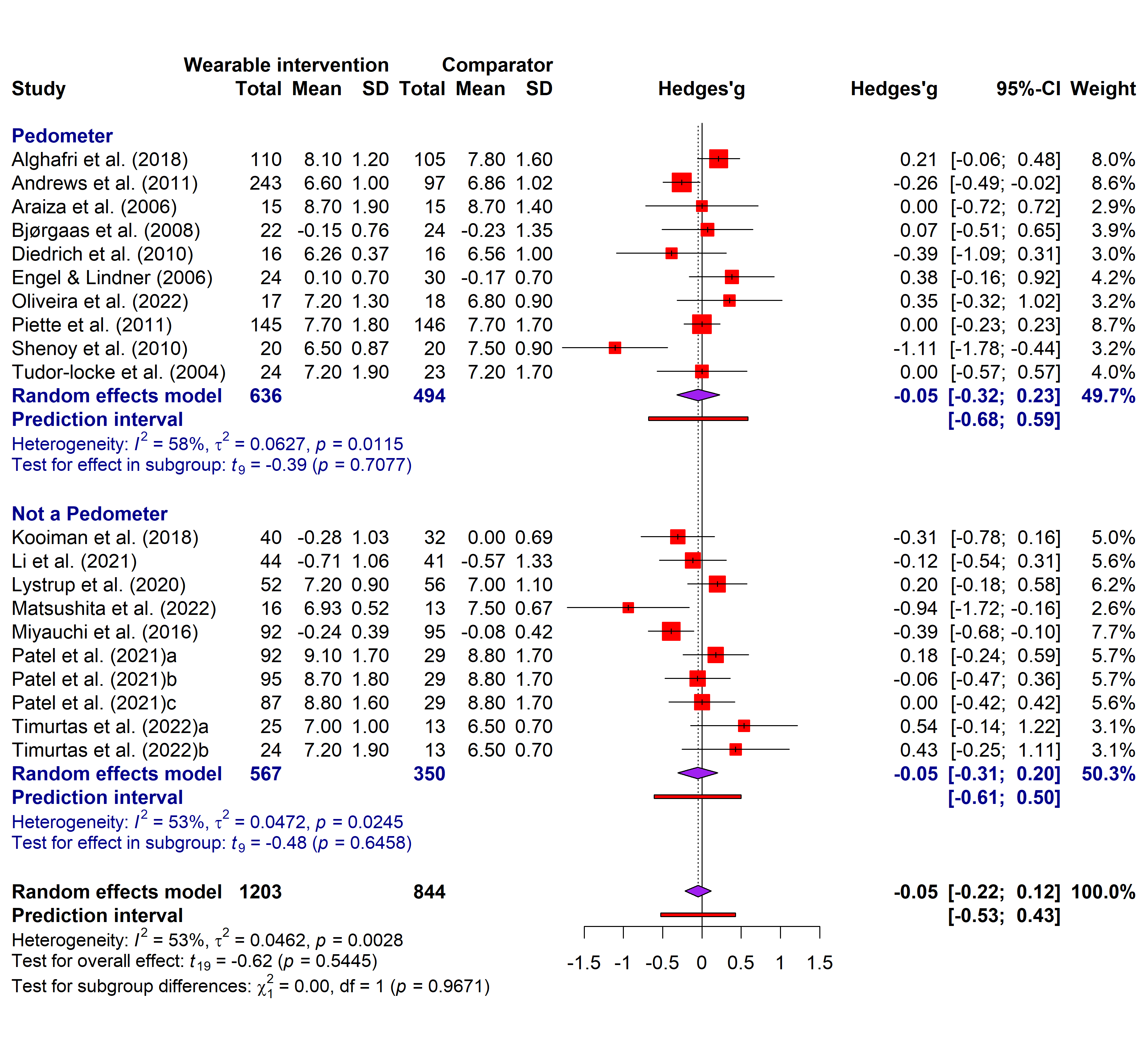


**SUPPLEMENTARY FIG. S14.** SUBGROUP ANALYSIS OF WEARABLE TECHNOLOGY-BASED PHYSICAL ACTIVITY INTERVENTIONS ON HBA1C BY TYPE OF WEARABLE.


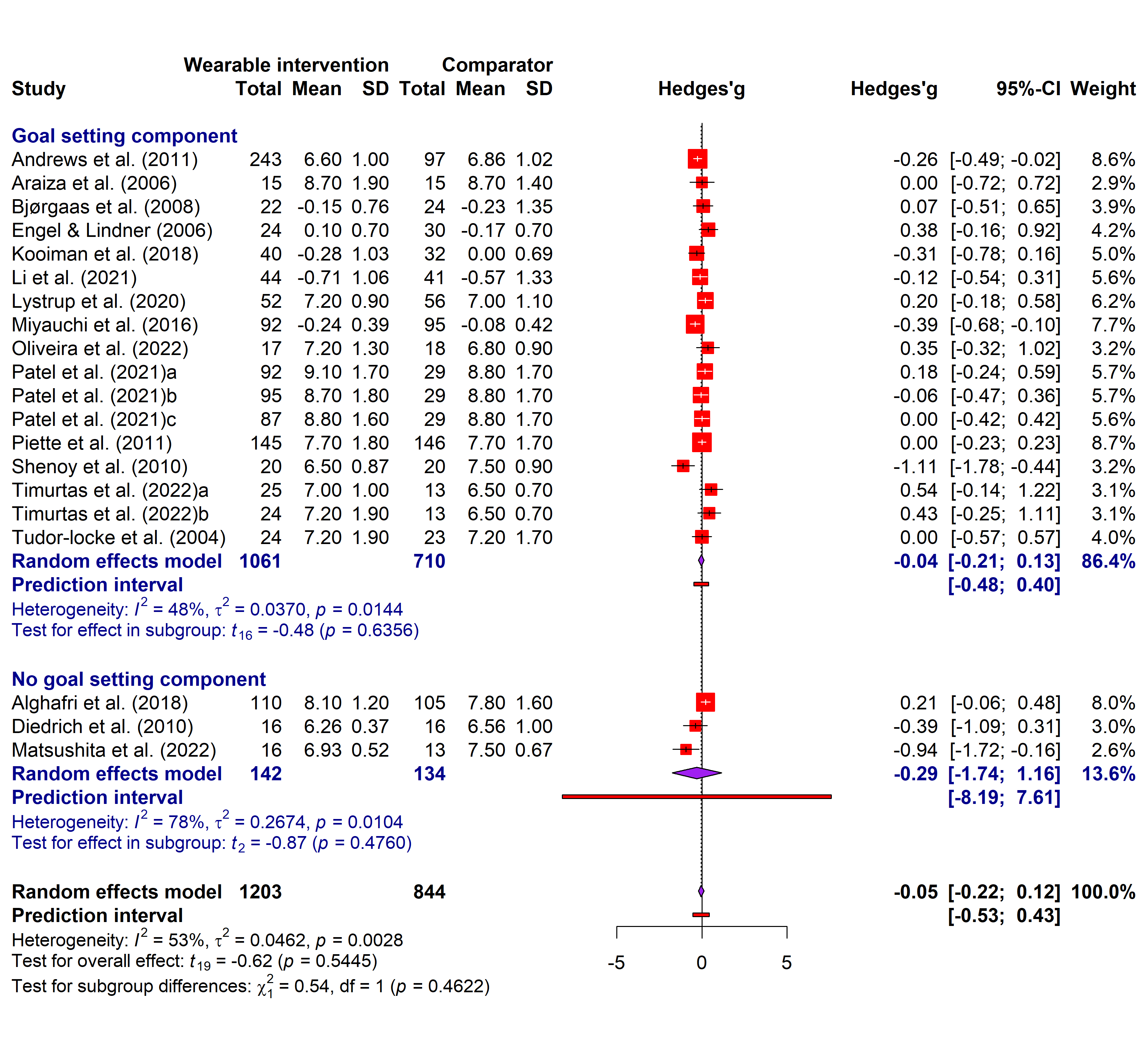


**SUPPLEMENTARY FIG. S15.** SUBGROUP ANALYSIS OF WEARABLE TECHNOLOGY-BASED PHYSICAL ACTIVITY INTERVENTIONS ON HBA1C GROUPED BY GOAL SETTING.


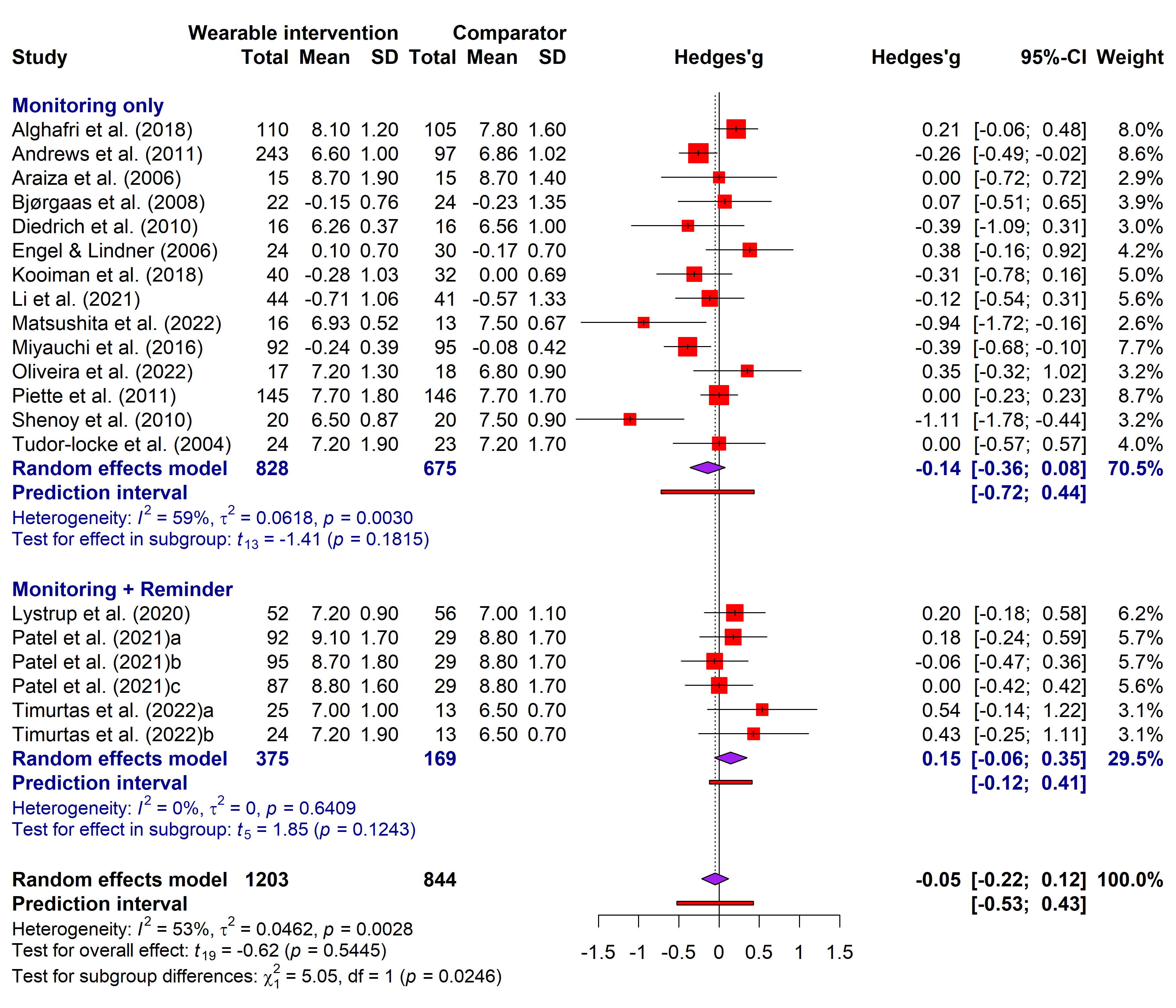


**SUPPLEMENTARY FIG. S16.** SUBGROUP ANALYSIS OF WEARABLE TECHNOLOGY-BASED PHYSICAL ACTIVITY INTERVENTIONS ON HBA1C GROUPED BY FUNCTION.


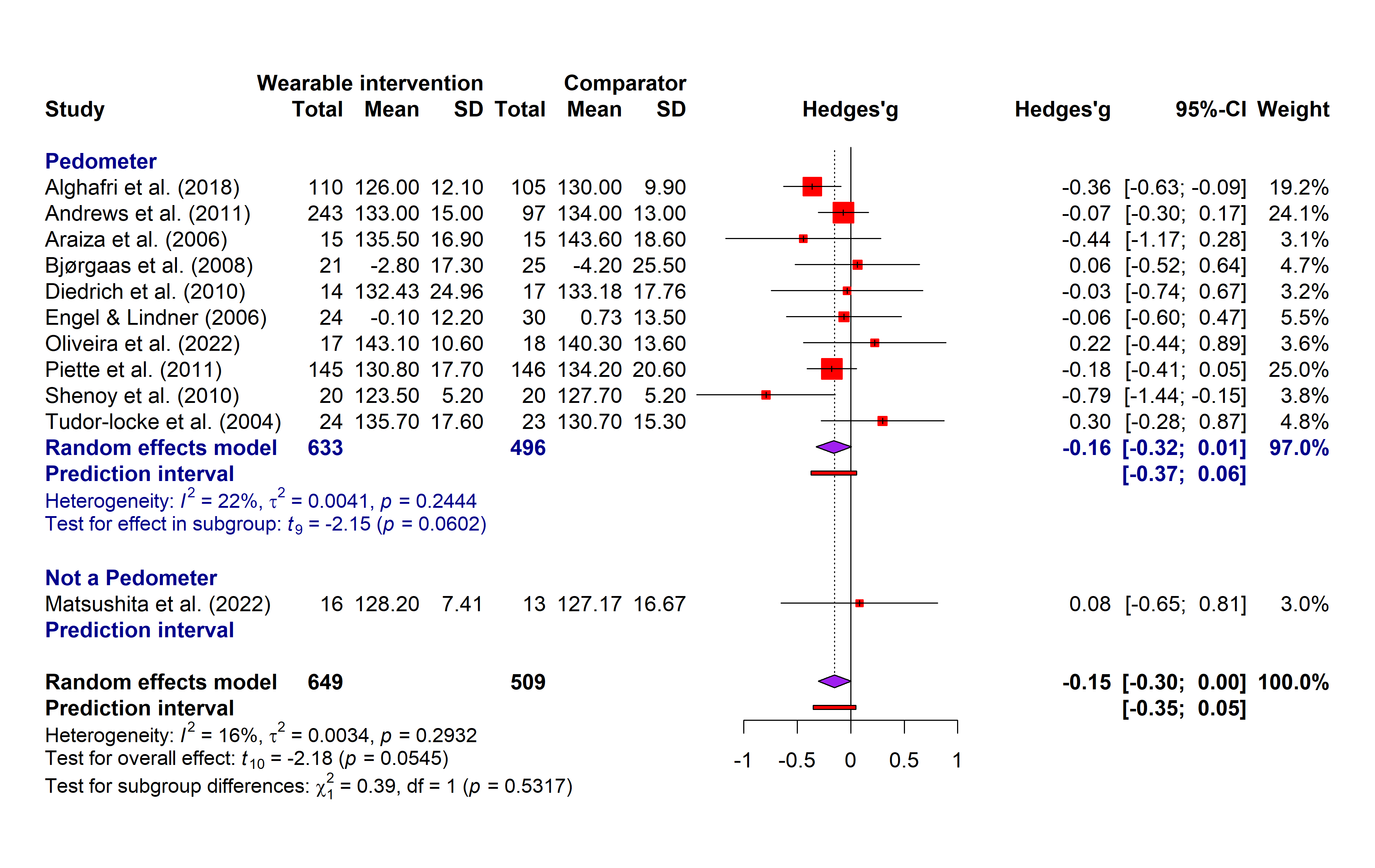


**SUPPLEMENTARY FIG. S17.** SUBGROUP ANALYSIS OF WEARABLE TECHNOLOGY-BASED PHYSICAL ACTIVITY INTERVENTIONS ON SYSTOLIC BLOOD PRESSURE BY TYPE OF WEARABLE.


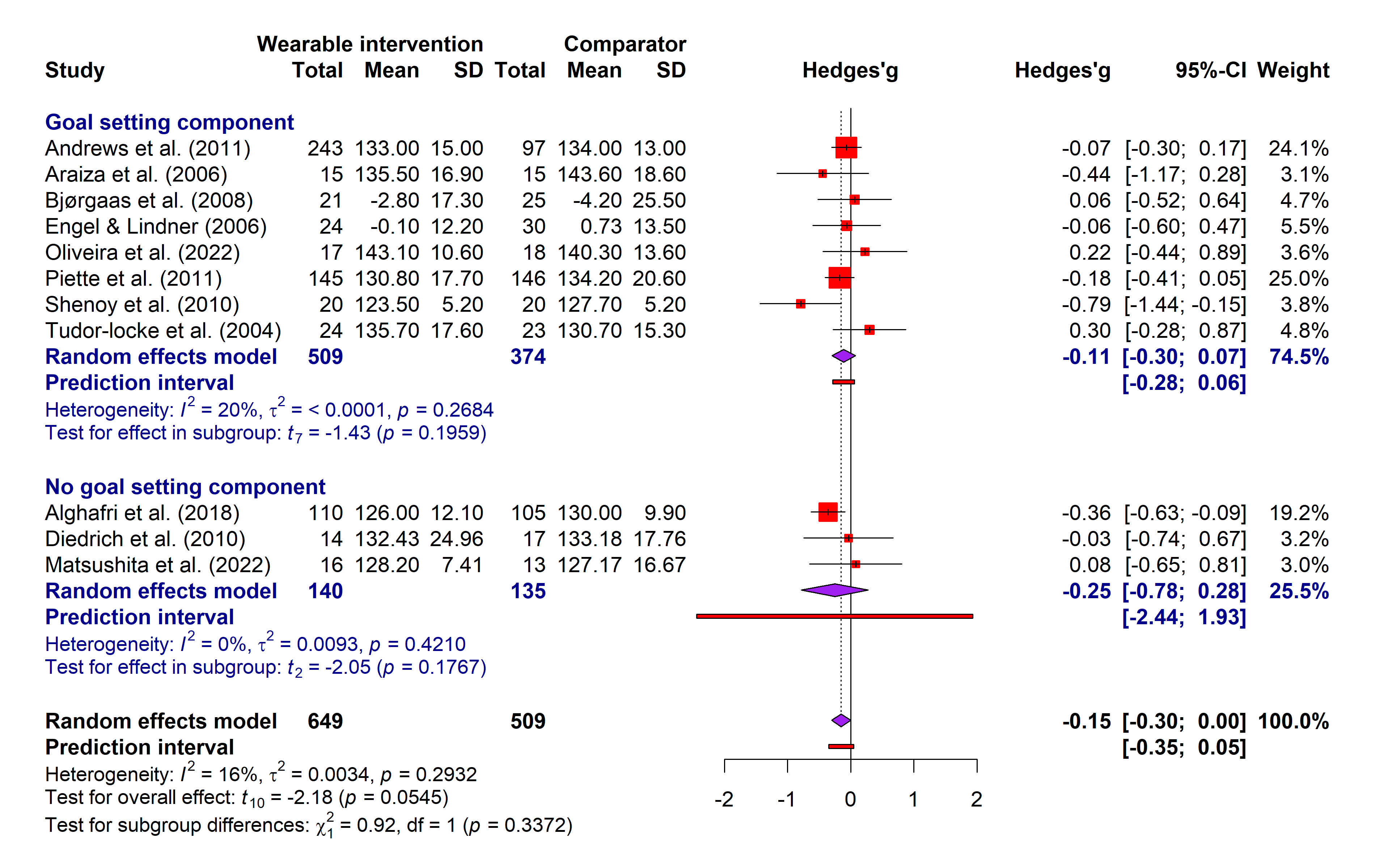


**SUPPLEMENTARY FIG. S18.** SUBGROUP ANALYSIS OF WEARABLE TECHNOLOGY-BASED PHYSICAL ACTIVITY INTERVENTIONS ON SYSTOLIC BLOOD PRESSURE BY GOAL SETTING.


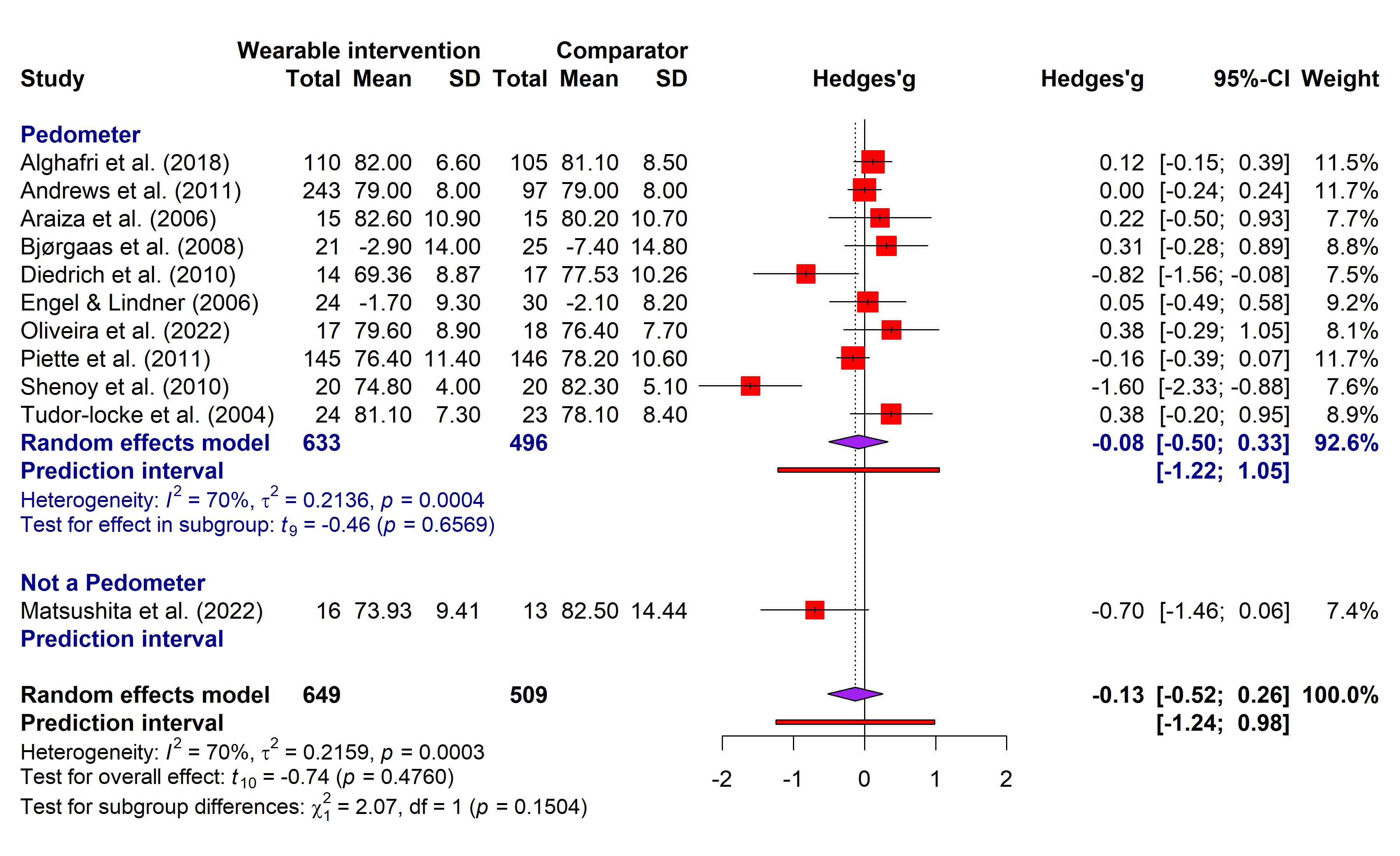


**SUPPLEMENTARY FIG. S19**. SUBGROUP ANALYSIS OF WEARABLE TECHNOLOGY-BASED PHYSICAL ACTIVITY INTERVENTIONS ON DIASTOLIC BLOOD PRESSURE BY TYPE OF WEARABLE.


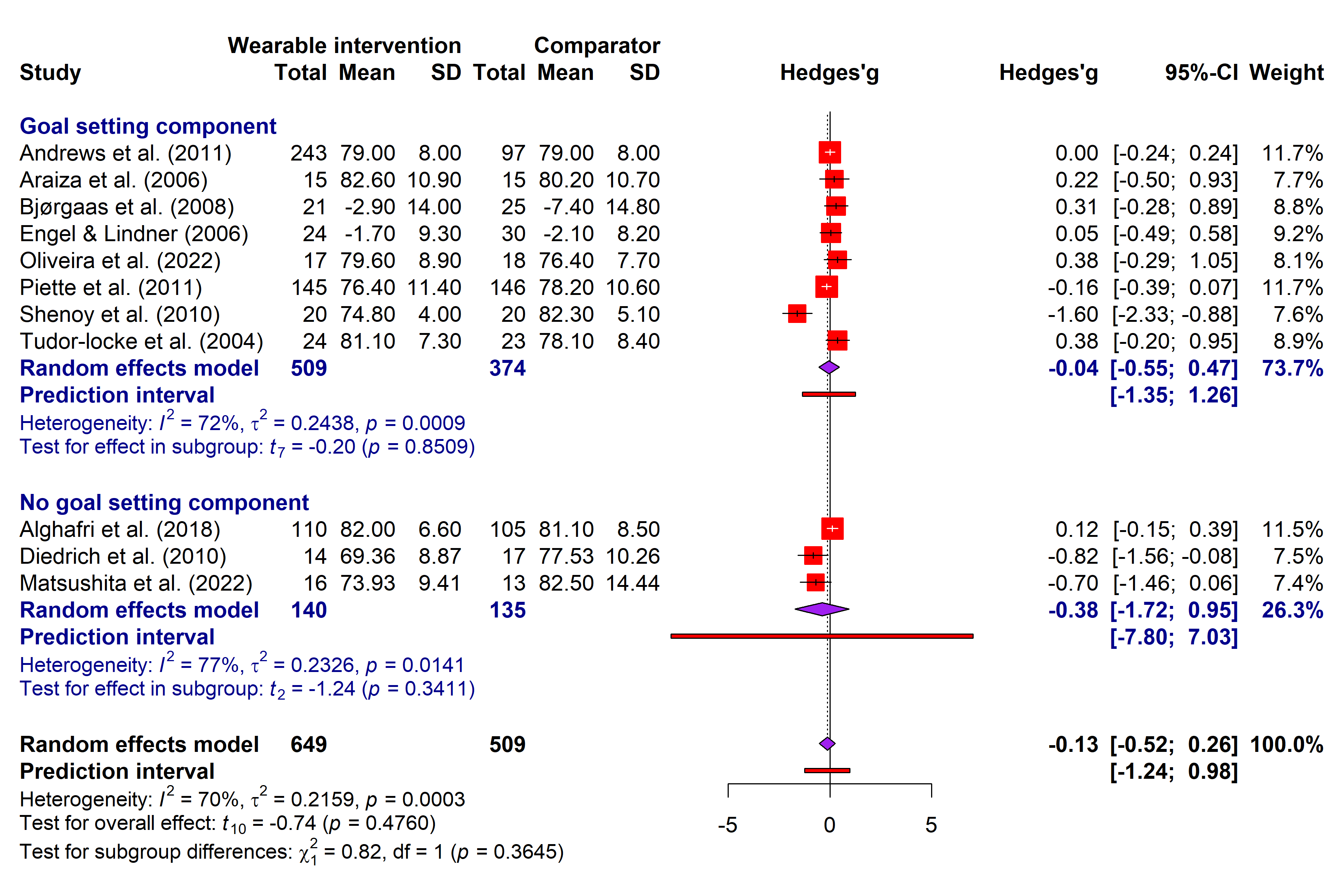


**SUPPLEMENTARY FIG. S20.** SUBGROUP ANALYSIS OF WEARABLE TECHNOLOGY-BASED PHYSICAL ACTIVITY INTERVENTIONS ON DIASTOLIC BLOOD PRESSURE BY GOAL SETTING.


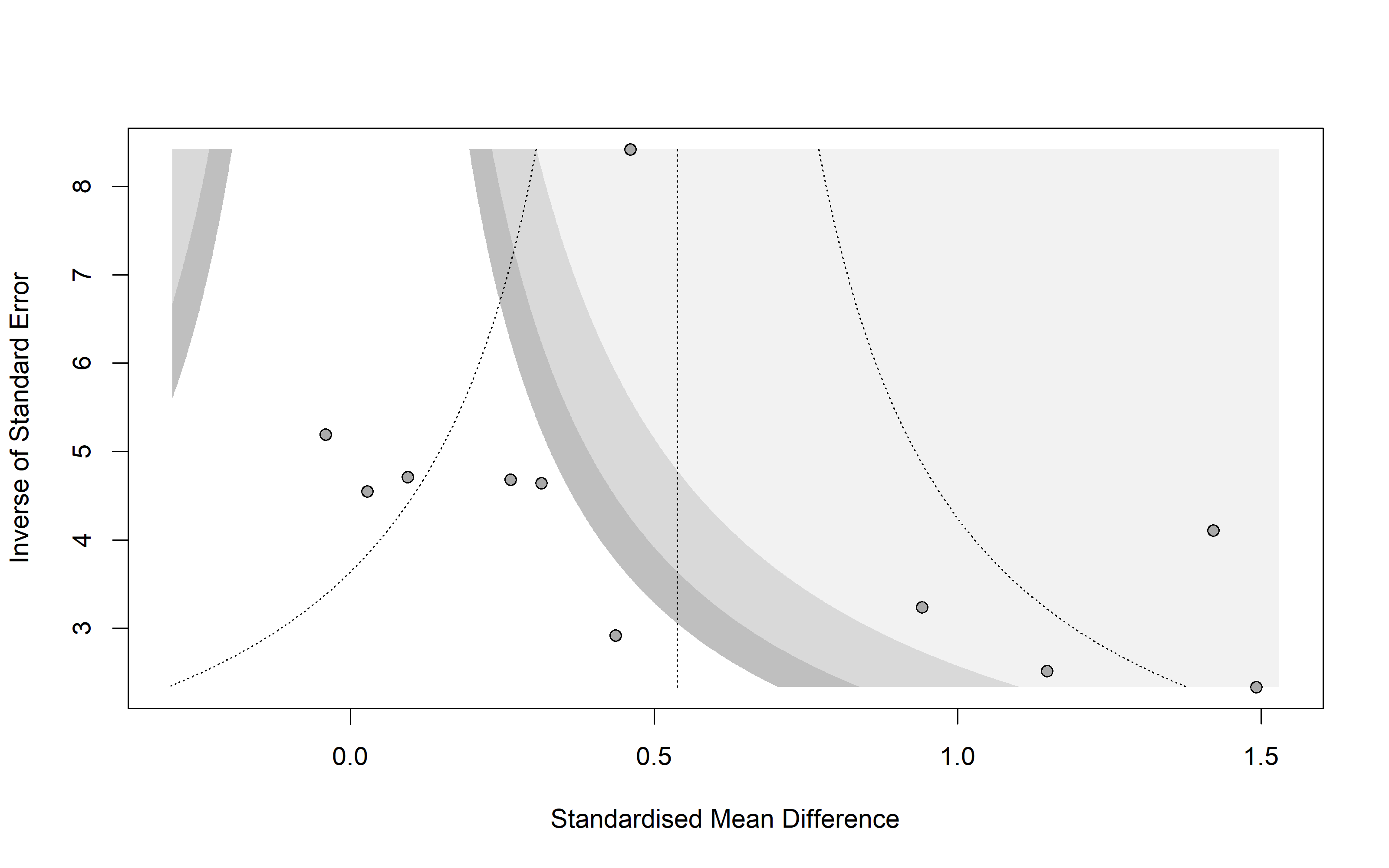


Linear regression test of funnel plot asymmetry

Test result: t = 1.37, df = 9, p-value = 0.2031

Sample estimates:

bias se.bias intercept se.intercept

2.3100 1.6830 -0.0414 0.3667

Details:

- multiplicative residual heterogeneity variance (tau^2 = 3.8148)

- predictor: standard error

- weight: inverse variance

- reference: Egger et al. (1997), BMJ

**SUPPLEMENTARY FIG. S21.** FUNNEL PLOT AND EGGER’S TEST FOR ASSESSMENT OF PUBLICATION BIAS ON STEPS PER DAY.


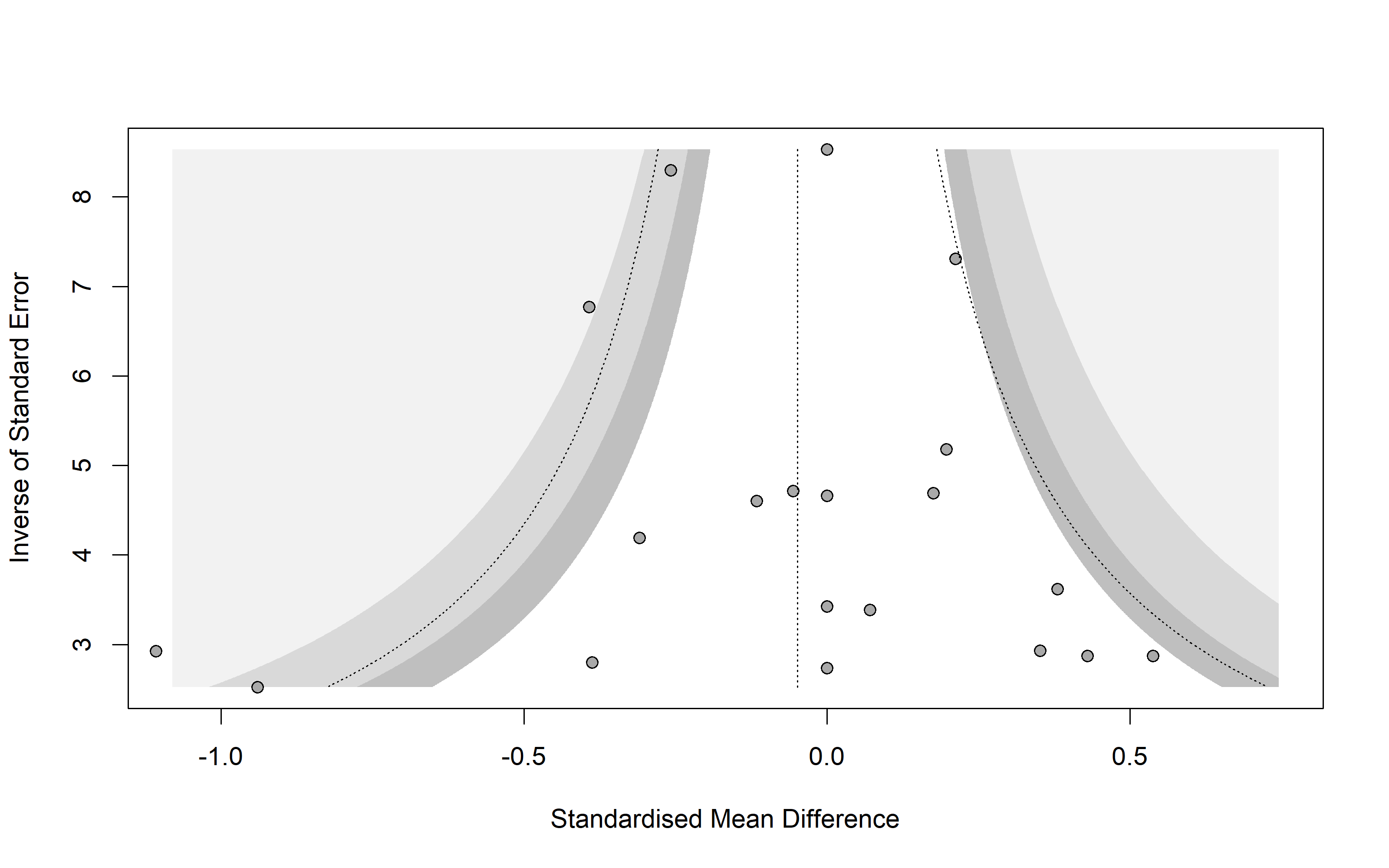


Linear regression test of funnel plot asymmetry

Test result: t = 0.09, df = 18, p-value = 0.9258

Sample estimates:

bias se.bias intercept se.intercept

0.0830 0.8788 -0.0723 0.1824

Details:

- multiplicative residual heterogeneity variance (tau^2 = 2.2516)

- predictor: standard error

- weight: inverse variance

- reference: Egger et al. (1997), BMJ

**SUPPLEMENTARY FIG. S22.** FUNNEL PLOT AND EGGER’S TEST FOR ASSESSMENT OF PUBLICATION BIAS ON HBA1C.


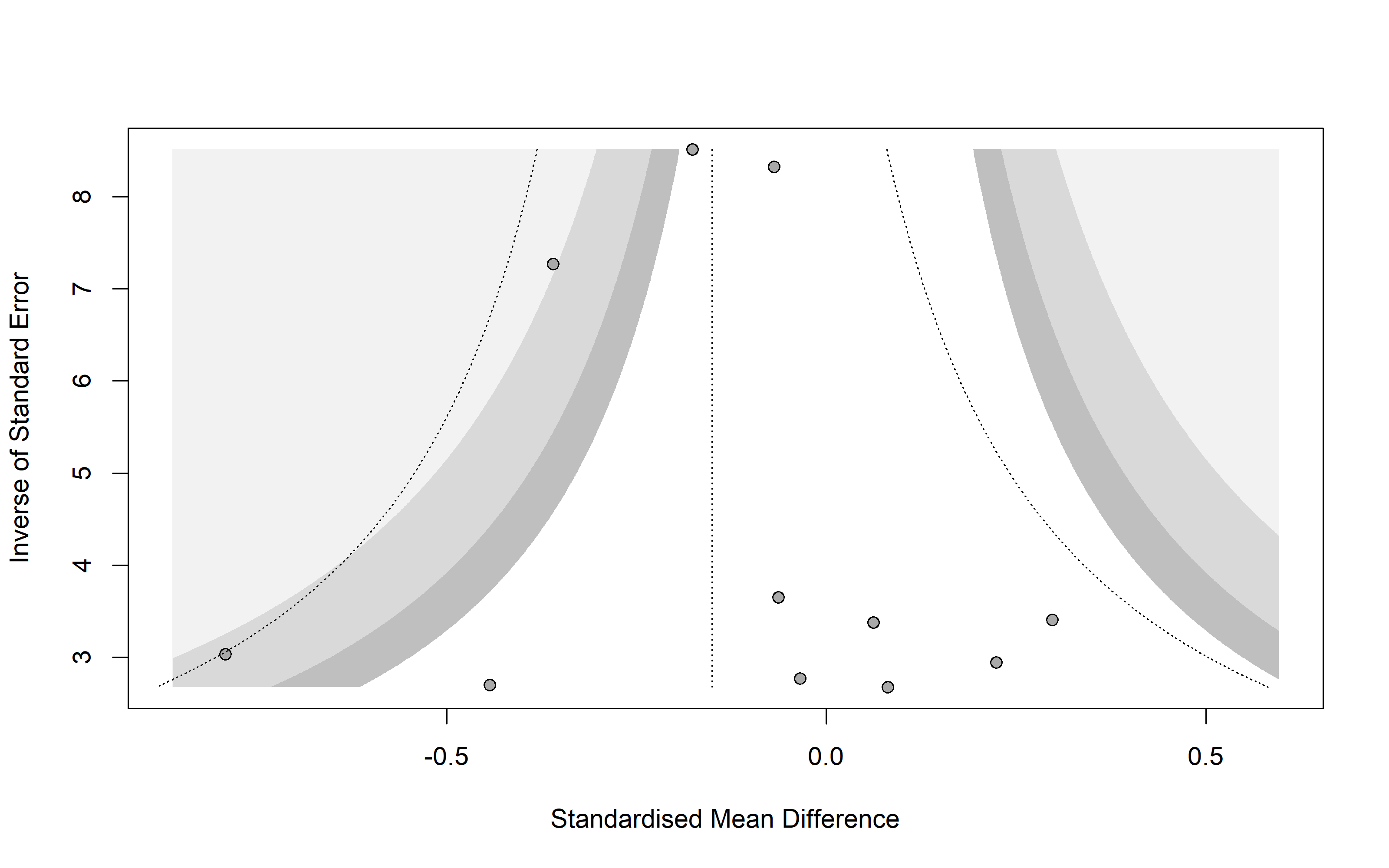


Linear regression test of funnel plot asymmetry

Test result: t = 0.55, df = 9, p-value = 0.5931

Sample estimates:

bias se.bias intercept se.intercept

0.4165 0.7518 -0.2281 0.1515

Details:

- multiplicative residual heterogeneity variance (tau^2 = 1.2765)

- predictor: standard error

- weight: inverse variance

- reference: Egger et al. (1997), BMJ

**SUPPLEMENTARY FIG. S23.** FUNNEL PLOT AND EGGER’S TEST FOR ASSESSMENT OF PUBLICATION BIAS ON SYSTOLIC BLOOD PRESSURE.


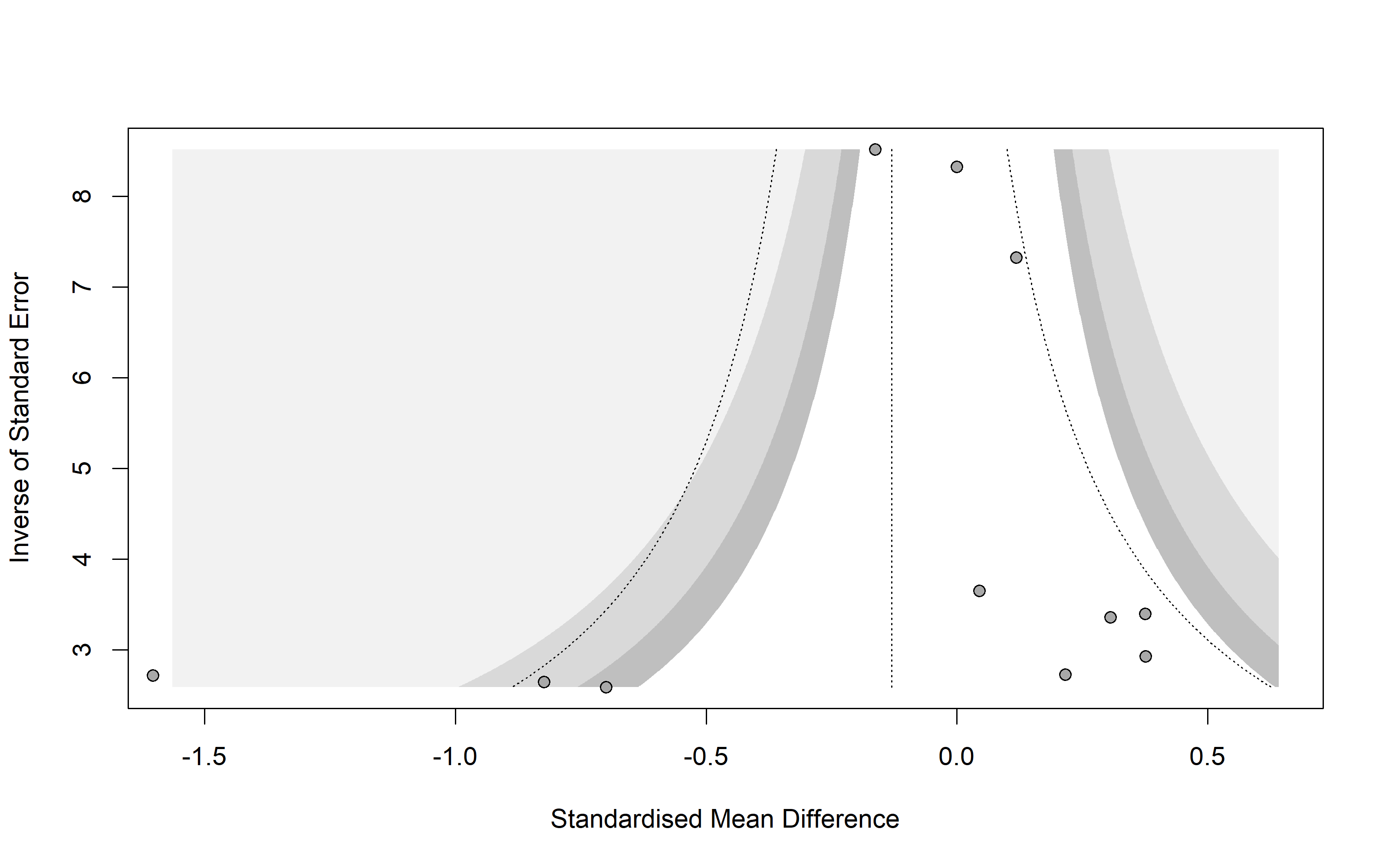


Linear regression test of funnel plot asymmetry

Test result: t = -0.73, df = 9, p-value = 0.4826

Sample estimates:

bias se.bias intercept se.intercept

-0.8890 1.2138 0.1052 0.2456

Details:

- multiplicative residual heterogeneity variance (tau^2 = 3.4795)

- predictor: standard error

- weight: inverse variance

- reference: Egger et al. (1997), BMJ

**SUPPLEMENTARY FIG. S24.** FUNNEL PLOT AND EGGER’S TEST FOR ASSESSMENT OF PUBLICATION BIAS ON DIASTOLIC BLOOD PRESSURE.
